# Natural compounds targeting glycolysis and TME in ovarian cancer: from metabolic crosstalk to therapeutic potential

**DOI:** 10.3389/fphar.2026.1877824

**Published:** 2026-08-18

**Authors:** Hongli Lv, Xi Jiang, Wei Zhang, Shuntian Kan, Yuan Zhang, Bo Wang, Fu Peng, Chenghao Yu

**Affiliations:** 1 Key Laboratory of Southwestern Chinese Medicine Resources, Department of Basic Medicine, Chengdu University of Traditional Chinese Medicine, Chengdu, China; 2 Key Laboratory of Drug-Targeting and Drug Delivery System of the Education Ministry, Sichuan Engineering Laboratory for Plant-Sourced Drug and Sichuan Research Center for Drug Precision Industrial Technology, West China School of Pharmacy, Sichuan University, Chengdu, China

**Keywords:** glycolysis, natural compounds, ovarian cancer, tumor microenvironment, Warburg effect

## Abstract

Ovarian cancer (OC) cells promote rapid proliferation *via* aerobic glycolysis and build an immunosuppressive microenvironment at the same time; therefore, this glycolytic pathway is now a potential drug target. Existing glycolysis inhibitors are limited by off-target toxicity, drug resistance, and variable efficacy across tumor subtypes. Natural compounds offer an alternative by modulating multiple glycolytic nodes, lowering lactate production, and reshaping the tumor microenvironment (TME). Therefore, this review summarizes 33 natural compounds that target glycolysis in OC. Their mechanisms of action focus on inhibiting glucose uptake, downregulating the PI3K/AKT/mTOR signaling pathway, suppressing the activities of glycolytic enzymes (HK2, PFK, PKM2, and LDHA) and HIF-1α transcription, reducing lactate production, ameliorating the TME, and ultimately inhibiting OC cell proliferation, migration, and invasion, thereby demonstrating the potential for developing novel anti-OC drugs. Clinical translation is still limited by inefficient extraction, low bioavailability, incomplete mechanistic characterization and a lack of clinical validation. Based on the current evidence and the aforementioned bottlenecks, this review offers a development path for natural glycolysis inhibitors in precision anti-OC therapy.

## 1 Introduction

Based on the statistics of 2023, cancer is the second biggest cause of death in the world after cardiovascular diseases (Force et al., 2025; An et al., 2026). Ovarian cancer (OC) is a rare and highly heterogeneous disease among malignant tumors. Due to the lack of atypical symptoms of OC, approximately 70% of cases are diagnosed at an advanced stage, and it is often accompanied by distant metastasis. Based on this, the prognosis for patients with this condition remains poor (Liu W. et al., 2019; Smolarz et al., 2025). Over the past decade, the incidence and mortality rates of OC have decreased, but it still ranks among the most malignant and deadly types of cancer (Xiao et al., 2017; Wentzensen et al., 2025). After conventional treatment, the 5-year overall survival rate for patients with advanced OC is only 10%–40% (Caruso et al., 2025). Over the past 5 years, more than 900,000 women worldwide have been diagnosed with OC. Approximately 70% of patients are diagnosed at an advanced stage. OC accounts for the largest share (91%) of the socioeconomic burden regarding the years of life lost. It is predicted that without more effective and comprehensive prevention and control measures, the cumulative number of deaths caused by OC worldwide will exceed 8 million between 2022 and 2050 (Hutchinson et al., 2025).

Glycolysis was the first metabolic pathway to be fully characterized (Chandel, 2021). In cancer cells, even if the oxygen supply is sufficient and the mitochondrial function is intact, aerobic glycolysis is still the primary mode of energy supply. This metabolic shift, characterized by increased glucose uptake and elevated lactate production rates, is known as the Warburg effect (Kooshan et al., 2024). Although glycolysis produces less adenosine triphosphate (ATP) than oxidative phosphorylation (OXPHOS), its efficiency can be nearly 100 times greater, which in turn promotes higher glycolytic flux (Locasale and Cantley, 2010; Koppenol et al., 2011). This metabolic reprogramming reflects a key adaptive change made by cancer cells in response to their increasing energy and biosynthetic demands. Studies have confirmed that glycolysis can promote tumor growth, metastasis, chemotherapy resistance, and inhibit tumor-cell apoptosis. Lactate is a product of glycolysis. Lactate accumulates within the tumor microenvironment (TME), altering the TME by lowering the extracellular pH and promoting immune suppression (Zhou D. et al., 2022; Lin T.-Y. et al., 2024). Key regulators and signaling pathways implicated in the orchestration of this cancer-specific metabolic reprogramming include hypoxia-inducible factor 1 alpha (HIF-1α), myelocytomatosis viral oncogene homolog (c-Myc), protein 53 (p53), hexokinase 2 (HK2), and lactate dehydrogenase A (LDHA), alongside the phosphatidylinositol 3-kinase (PI3K)/protein kinase B (AKT)/mammalian target of rapamycin (mTOR) axis (Chelakkot et al., 2023; Li et al., 2023).

Increased glycolysis also contributes to the remodeling of the OC microenvironment in the form of continuous export of lactate. Extracellular lactate acts as a signaling metabolite that supports cancer-cell survival, alters epigenetic regulation, and suppresses antitumor immunity (Jin B. et al., 2025). Stromal acidification supports matrix degradation and epithelial–mesenchymal transition (EMT), which leads to higher invasion and metastasis of tumors (Kenny et al., 2008). The lactate shuttle between cancer-associated fibroblasts (CAFs) and OC cells further establishes a metabolic symbiosis that supports tumor progression (Lisanti et al., 2013; Suh et al., 2014). OC glycolysis can maintain malignant proliferation, promote immune suppression, and create a pro-metastatic niche.

In OC cells, aerobic glycolysis serves to fuel rapid cell proliferation, while also contributing to the formation of an immunosuppressive microenvironment. This distinct metabolic dependence makes glycolysis an appealing target for intervention. Currently, targeting glycolysis drugs have many defects, including off-target toxicity, acquired resistance, and variable efficacy across different subtypes of OC (Kozal et al., 2021; Li et al., 2026). Natural products derived from plants, microorganisms, marine organisms, and other biological resources offer an appealing alternative (Katz and Baltz, 2016; Zhang et al., 2020; Chopra and Dhingra, 2021). After thousands of years of evolution, the chemical structure of natural compounds is very distinct, and its complexity often breaks through the limitations of artificial design. Natural compounds can exhibit strong biological activity at low concentrations. Compared to some synthetic drugs, these compounds typically exhibit superior efficacy, safety, and tolerability. This is closely related to their unique mechanism of multi-target synergistic action (Quintero-Rincón et al., 2025; Scotti and Scotti, 2025; Hashemi et al., 2026). Targeted therapy based on natural compounds that simultaneously modulate multiple glycolytic nodes, reshape the TME, and overcome chemoresistance represents a highly promising cancer-management strategy, thereby garnering considerable interest in the treatment of OC.

There is no comprehensive review on the targeted glycolysis and the TME of OC in the research of natural products. Various compounds targeting glycolysis and lactate-mediated immunosuppression have been discovered in existing studies. However, the mechanisms of these compounds are scattered, and the metabolic–immune regulatory networks have not been elucidated clearly. There is an urgent need to explore how natural compounds regulate glycolysis and the TME systematically in OC and predict their potential.

This review systematically summarizes 33 natural compounds that can regulate glycolysis and remodel the TME in OC. The reported mechanisms include curtailing glucose uptake, dampening PI3K/AKT/mTOR signaling, and suppressing the expression or activity of glycolytic enzymes (HK2, phosphofructokinase (PFK), pyruvate kinase isozyme type M2 (PKM2), and LDHA) and the transcription factor HIF-1α. These effects inhibit lactate production and export, decrease the level of extracellular acidification in the TME, and relieve lactate-induced immunosuppression. These compounds can inhibit he proliferation, migration, and invasion of OC cells. Furthermore, they are expected to restore the body’s antitumor immunity by normalizing the TME. These findings highlight the broad therapeutic promise of natural glycolysis inhibitors as novel anti-OC drug candidates.

## 2 Glycolysis in ovarian cancer

**FIGURE 1:**
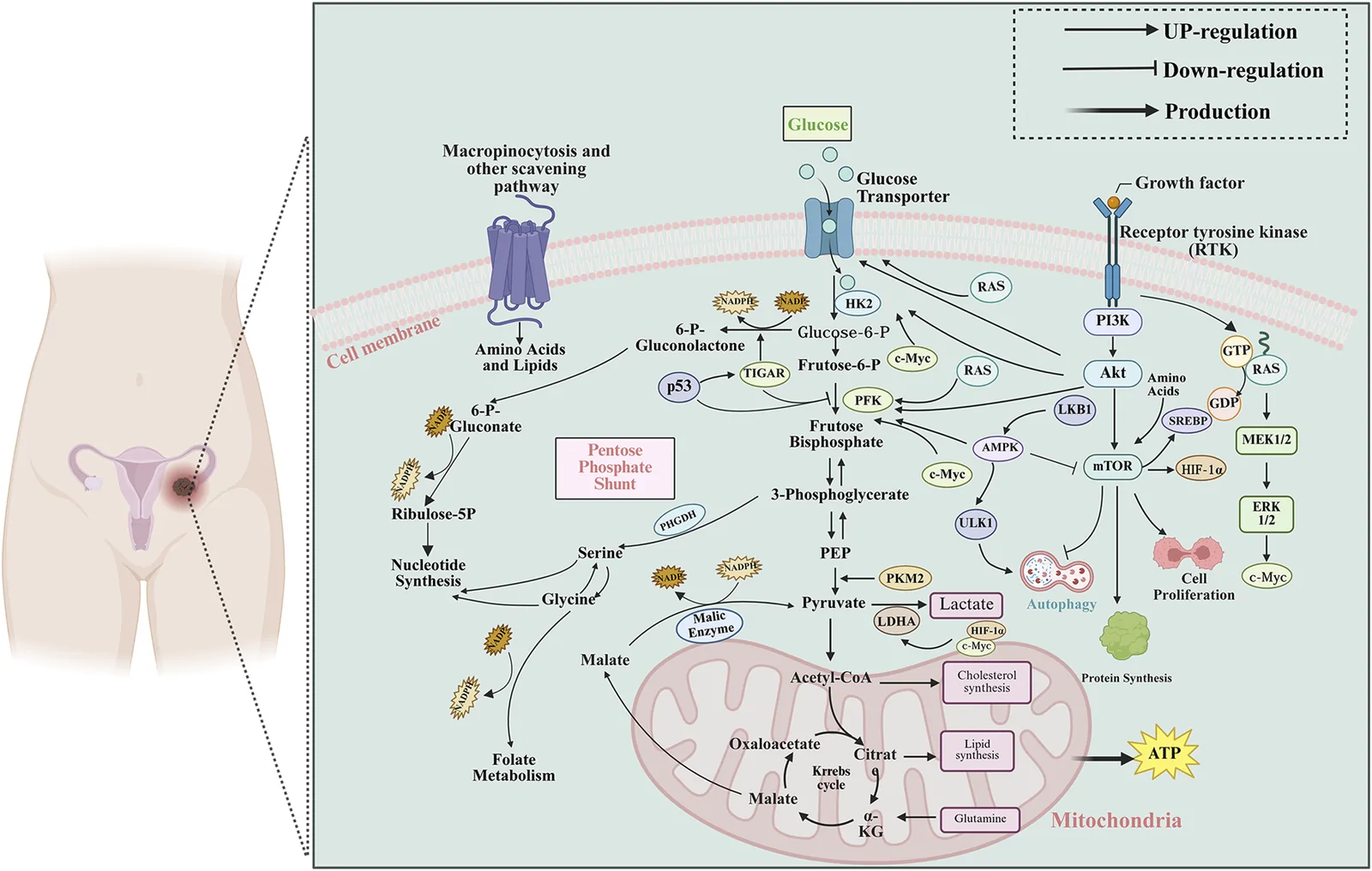
Diagram of glycolysis-related mechanisms in ovarian cancer. Growth factor-stimulated RTKs signal through the RAS–PI3K–AKT–mTOR and RAS–MEK–ERK cascades, cooperating with c-Myc and HIF-1α to upregulate glucose transporters and key glycolytic enzymes. Mitochondria-bound HK2 initiates glucose phosphorylation; PFKFB3-generated fructose-2,6-bisphosphate activates PFK-1; attenuated PKM2 diverts phosphoenolpyruvate into biosynthetic routes; and LDHA produces lactate while regenerating NAD^+^. Glycolytic intermediates supply the pentose phosphate pathway for nucleotide synthesis and the serine synthesis pathway *via* PHGDH. Citrate exported from mitochondria provides acetyl-CoA for lipid and cholesterol synthesis. Loss of wild-type p53 abolishes TIGAR-mediated glycolytic suppression. Impaired LKB1–AMPK signaling disinhibits mTOR/SREBP and reduces ULK1-driven autophagy, increasing dependence on macropinocytosis for amino acids and lipids. Glutamine anaplerosis sustains the TCA cycle. Collectively, this reprogrammed network fuels rapid proliferation, macromolecular biosynthesis, and an acidic, lactate-rich microenvironment that promotes immune evasion. Created in https://BioRender.com. Abbreviations: 6-P-Gluconolactone, 6-phosphogluconolactone; α-KG, alpha-ketoglutarate; Acetyl-CoA, acetyl coenzyme A; Akt, protein kinase B; AMPK, adenosine monophosphate-activated protein kinase; c-Myc, myelocytomatosis viral oncogene homolog; ERK1/2, extracellular signal-regulated kinase 1/2; Fructose-6-p, fructose-6-phosphate; GDP, guanosine diphosphate; Glucose-6-P, glucose-6-phosphate; GTP, guanosine triphosphate; HIF-1α, hypoxia-inducible factor 1 alpha; HK2, hexokinase 2; LDHA, lactate dehydrogenase A; LKB1, liver kinase B1; MEK1/2, mitogen-activated protein kinase 1/2; mTOR, mammalian target of rapamycin; NADP, nicotinamide adenine dinucleotide phosphate; NADPH, nicotinamide adenine dinucleotide phosphate hydrogen; p53, protein 53; PEP, phosphoenolpyruvate; PFK, phosphofructokinase; PI3K, phosphatidylinositol 3-kinase; PKM2, pyruvate kinase isozyme type M2; RAS, rat sarcoma virus; SREBP, sterol regulatory element-binding protein; TIGAR, TP53-induced glycolysis and apoptosis regulator; ULK1, unc-51-like autophagy activating kinase 1.

Tumor cells preferentially utilize glycolysis, the conversion of glucose to lactate, rather than aerobic oxidation (OXPHOS), the main metabolic pathway in healthy cells, to generate energy even under normal oxygen levels. In OC, metabolic reprogramming is not an inefficient use of energy; rather, it is a fundamental adaptation to enable rapid proliferation, survival, and invasion of cancer cells and drug resistance (Ganapathy-Kanniappan and Geschwind, 2013; Lu et al., 2015; Chelakkot et al., 2023). The reprogramming of metabolism is mediated through several pathways that are elaborated below (Figure 1).

### 2.1 Regulation of oncogenes and tumor-suppressor genes and dysregulated activation of signaling pathways

#### 2.1.1 PI3K/AKT/mTOR

The PI3K/AKT/mTOR signaling pathway plays a major role in cell growth, proliferation, and metabolism, and it is often dysregulated in OC (Ediriweera et al., 2019; Ghoneum and Said, 2019; Van Der Ploeg et al., 2021). AKT directly phosphorylates and activates a range of glycolytic enzymes such as HK2 and 6-phosphofructo-2-kinase/fructose-2,6-bisphosphatase 2 (PFKFB2). AKT also promotes the translocation of glucose transporter 1 (GLUT1) to the cell membrane, leading to increased glucose uptake. mTOR acts as a key regulatory node, upregulating the transcription of several glycolysis-related genes, such as HK2, and promoting the synthesis of proteins to support the rapid proliferation of cancer cells.

#### 2.1.2 HIF-1α

Ovarian tumors are often characterized by hypoxic areas, but HIF-1α can remain stable even under normoxic conditions due to genetic mutations or activation of signaling pathways such as PI3K/AKT. HIF-1α functions as a master regulator that directly drives the transcription of nearly all glycolysis-related genes (Chen et al., 2022a; Wu H. et al., 2023; Fontana et al., 2024; Mori et al., 2024). These include genes encoding GLUT1 and glucose transporter 3 (GLUT3), which enhance glucose uptake; hexokinase 1 (HK1) and HK2, which catalyze the first step of glycolysis; and 6-phosphofructo-2-kinase/fructose-2,6-bisphosphatase 3 (PFKFB3), which generates the potent allosteric activator fructose-2,6-bisphosphate (F-2,6-BP), thus significantly enhancing phosphofructokinase-1 (PFK-1) activity. HIF-1α also upregulates the expression of LDHA, which catalyzes the conversion of pyruvate to lactate while regenerating the coenzyme nicotinamide adenine dinucleotide (NAD+), thereby enabling glycolysis to proceed continuously.

#### 2.1.3 c-Myc

c-Myc is an important oncogene that is aberrantly highly expressed in multiple cancers. It synergistically interacts with HIF-1α, jointly promoting the expression of several glycolytic genes, including those encoding GLUT1, HK2, enolase 1 (ENO1), and LDHA (Ma et al., 2018; Liu M. et al., 2020).

#### 2.1.4 p53

Wild-type p53 suppresses glycolysis and promotes aerobic oxidation, primarily by downregulating the expression of GLUT1 and glucose transporter 4 (GLUT4) and upregulating that of TP53-induced glycolysis and apoptosis regulator (TIGAR). However, p53 is mutated in over half of OC cases, leading to the loss of this inhibitory effect and the subsequent enhancement of glycolysis (Madan et al., 2019; Tiwari et al., 2020; Alvarado-Ortiz et al., 2021; Wallis et al., 2023; Wang C.-K. et al., 2023).

### 2.2 Abnormal expression of key glycolytic enzymes

#### 2.2.1 HK2

HK2 catalyzes the initial step of glucose metabolism and is highly expressed in OC. This enzyme binds to voltage-dependent anion channels on the outer membrane of mitochondria, allowing it to first acquire ATP produced by mitochondria, which is used to catalyze the reaction and escape the inhibition of apoptosis signals (Xue et al., 2019; Li et al., 2025).

#### 2.2.2 PFK-1 and its regulatory factor PFKFB3

PFK-1 is the most critical rate-limiting enzyme in glycolysis. PFKFB3 activates the activity of PFK-1 and synthesizes F-2,6-BP. The high expression of PFKFB3 in OC significantly accelerates glycolytic flux, effectively acting as a metabolic “accelerator” (Xintaropoulou et al., 2018; Jiang et al., 2022; Xiao et al., 2025).

#### 2.2.3 PKM2

In OC, low-activity PKM2 dimers are dominant. These dimers slow down the final step of glycolysis and divert upstream intermediate metabolites into biosynthetic pathways (pentose phosphate pathway and serine synthesis), thereby supporting the rapid proliferation of cells. The nuclear translocation of dimeric PKM2, where it acts as a protein kinase and transcriptional co-activator to upregulate genes involved in glycolysis, lipid metabolism, and cell-cycle progression, directly promotes the Warburg effect, hypoxia survival, metastasis, and chemoresistance (Zhu et al., 2021; Zhou S. et al., 2022; Zhang J. et al., 2024).

#### 2.2.4 LDHA

LDHA catalyzes the final step of glycolysis, which is the conversion of pyruvate to lactate. Increased LDHA expression not only maintains glycolytic flux but also generates lactate, an important signaling molecule and immunomodulator. This lactate acidifies the TME, which inhibits T-cell activity and supports immune evasion by cancer cells (Cong et al., 2024; Liu D. et al., 2024).

Collectively, the aberrant activation of PI3K/AKT/mTOR, HIF-1α, and c-Myc, together with the loss of the p53 function, upregulates a battery of glycolytic enzymes (HK2, PFKFB3, PKM2, and LDHA) in OC cells. This not only fuels rapid ATP generation and biosynthesis but also sets the stage for profound remodeling of the tumor microenvironment, as discussed in the next section.

## 3 Glycolysis-driven remodeling of the tumor microenvironment in ovarian cancer

**FIGURE 2:**
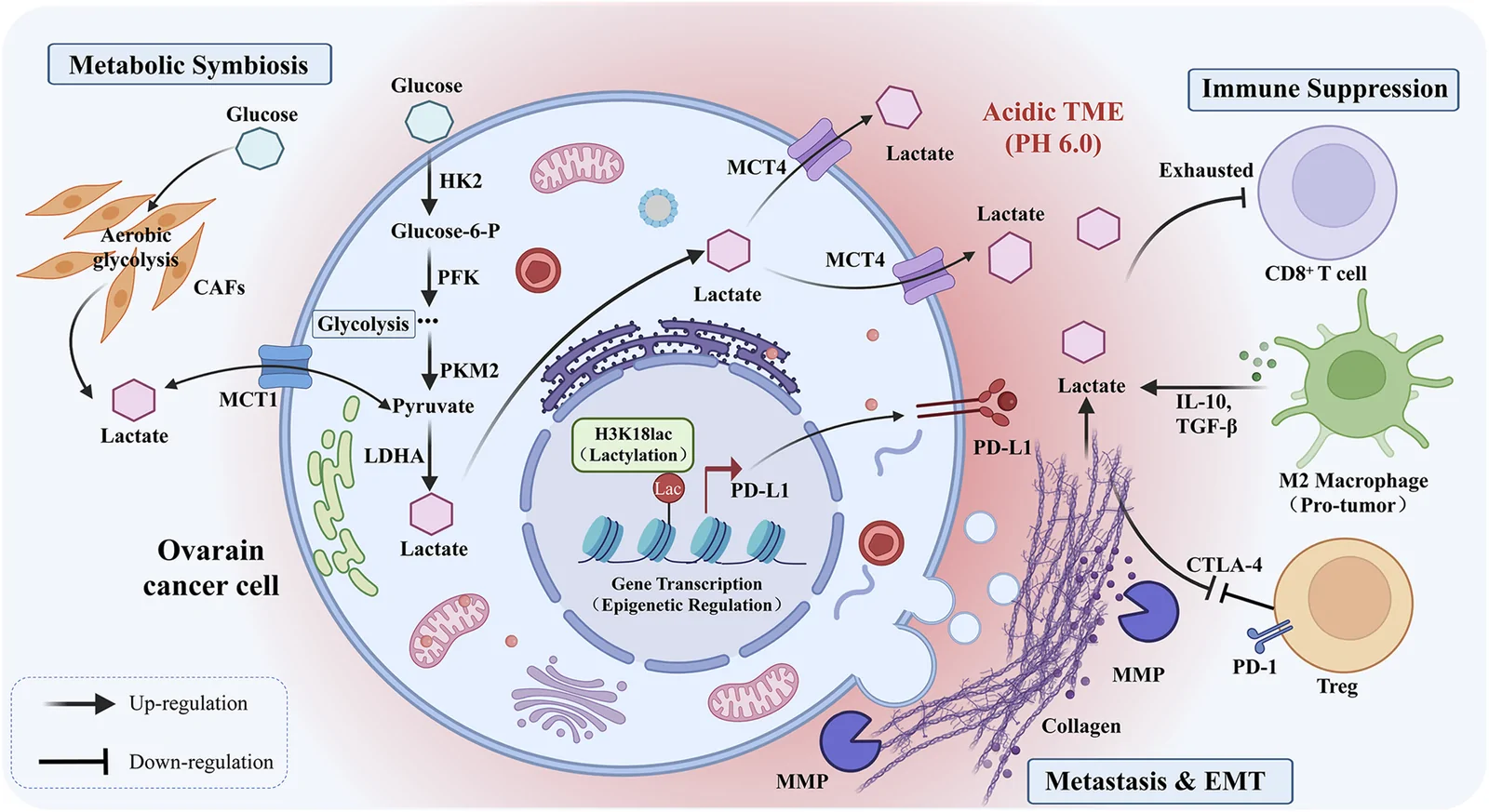
Glycolysis-driven tumor microenvironment remodeling in ovarian cancer. CAFs undergo TGF-β-induced aerobic glycolysis and export lactate *via* MCT4; cancer cells import it through MCT1 to fuel OXPHOS (reverse Warburg effect). Cancer cell-intrinsic glycolysis generates lactate exported *via* MCT4, acidifying the TME and driving H3K18 lactylation to upregulate PD-L1. Lactate and low pH promote M2-like macrophage polarization, impair CD8^+^ T cells, and induce EMT and invasion through MMP activation and Snail/Twist stabilization. This metabolic crosstalk establishes an immunosuppressive, pro-metastatic niche. Created in https://BioRender.com. Abbreviations: CAFs, cancer-associated fibroblasts; CD8+T cell, cluster of differentiation 8 positive T cell; CTLA-4, cytotoxic T-lymphocyte associated protein 4; EMT, epithelial-mesenchymal transition; Glucose-6-P, glucose-6-phosphate; HK2, hexokinase 2; IL-10, interleukin-10; LDHA, lactate dehydrogenase A; M2 Macrophage, M2-type macrophage; MCT1, monocarboxylate transporter 1; MCT4, monocarboxylate transporter 4; MMP, mitochondrial membrane potential; PD-1, programmed cell-death protein 1; PD-L1, programmed death-ligand 1; PFK, phosphofructokinase; PKM2, pyruvate kinase isozyme type M2; TGF-β, transforming growth factor-beta; TME, tumor microenvironment.

Metabolic shift toward glycolysis in OC cells generates a significant lactate burden with extracellular consequences. This excess lactate is actively exported to the tumor stroma, where it accumulates and modifies the physicochemical and immunological properties of the surrounding microenvironment, rather than being confined to the cytosol (Yu et al., 2021). Lactate functions simultaneously as a metabolic substrate and a signaling mediator, driving the structural and functional remodeling characteristic of the OC milieu (Figure 2).

### 3.1 Lactate as a pleiotropic oncometabolite in the OC milieu

Lactate was historically considered a metabolic waste product. It is now recognized as a multifunctional metabolite that influences both OC cells and the TME. In OC, glycolytic flux remains elevated under normoxic conditions. Lactate is mainly exported by monocarboxylate transporter 4 (MCT4), which is overexpressed in OC and correlates with worse progression-free survival (Chatterjee et al., 2023). Extracellular lactate concentrations in poorly perfused tumor areas can reach 10 mM–30 mM (Harjes, 2017).

Lactate signaling occurs through GPR81 (HCAR1, G protein-coupled receptor 81), a Gαi-coupled receptor that is widely expressed in OC tissues (Kerslake et al., 2022). Extracellular lactate activation of GPR81 enhances autophagy in cancer cells, contributing to cell survival under metabolic stress (Lundø et al., 2020), and it also drives malignant phenotypes and chemoresistance in OC (Zheng et al., 2025). Lactate also serves as a metabolic substrate. Cancer cells take up lactate *via* monocarboxylate transporter 1 (MCT1), convert it to pyruvate through lactate dehydrogenase B (LDHB), and use it to fuel the tricarboxylic acid (TCA) cycle and OXPHOS (Sun C. et al., 2020). This spares glucose for biosynthetic pathways.

### 3.2 Histone lactylation as an epigenetic link between glycolysis and gene regulation

Histone lysine lactylation (Kla) is identified as a post-translational modification that is driven by lactate-derived lactyl CoA (Zhang D. et al., 2019). In OC, enhanced glycolysis increases intracellular lactate and promotes histone lactylation, especially at the site of histone H3 lysine 18 lactylation (H3K18la). There are several promoters of genes that promote cell proliferation, DNA damage repair, and immune regulation containing this mark (Gao et al., 2025; Sun et al., 2025). Reduction of glycolytic lactate production lowers H3K18la and attenuates lactate-responsive gene expression.

### 3.3 Acidosis-induced suppression of antitumor immunity

Lactate export acidifies the TME to pH 6.0–6.5 (De La Cruz-López et al., 2019). In OC, this impairs CD8^+^ T cells (reduced interferon-γ, increased programmed cell-death protein 1/T-cell immunoglobulin and mucin-domain containing-3 (TIM-3)) and natural killer (NK) cell cytotoxicity (Brand et al., 2016; Liu K. et al., 2024). Regulatory T-cells (Tregs) are relatively resistant, shifting the Treg/CD8^+^ ratio toward immunosuppression (Chen et al., 2022b).

Lactate can also modify the function of immune evasion at the level of epigenetics and immune-metabolism, in addition to lowering the pH. Lactate promotes the polarization of M2-like tumor-associated macrophages (arginase-1 and IL-10) and expands myeloid-derived suppressor cells (MDSCs) (Komura et al., 2020). Lactate promotes PD-L1 by regulating H3K18 lactylation at the PD-L1 promoter; this process requires LDHB, and therefore, knockdown of LDHB reduces H3K18la occupancy, suppresses PD-L1 transcription, and enhances T-cell cytotoxicity (Hu et al., 2025). At the same time, lactate promotes M2 polarization in macrophages *via* H3K18la-mediated upregulation of the C–C-motif chemokine ligand 18 (CCL18), and lactate-treated macrophages promote OC cell proliferation and migration through the G protein-coupled receptor 132 (Gpr132)–CCL18 axis (Sun J. et al., 2024). Lactate can also induce CD8^+^ T-cell exhaustion in high-grade serous OC *via* forkhead box K1 (FOXK1)-driven glycolipid metabolism and thymocyte selection-associated high mobility group box protein (TOX)-induced histone lactylation; thus, these mechanisms impair T-cell effector functions (Li, 2026). Collectively, the above results show that lactate acts as a central node connecting glycolysis to the induction of immune checkpoint expression, macrophage reprogramming, and T-cell exhaustion, thus promoting an immunosuppressive network in the OC microenvironment.

### 3.4 Acidity and the roles of invasion, metastasis, and chemoresistance

Low extracellular pH activates matrix metalloproteinase-2 (MMP-2) and matrix metalloproteinase-9 (MMP-9), which degrade the extracellular matrix (ECM) and promote peritoneal dissemination in OC (Kenny et al., 2008). Acidosis directly promotes EMT by increasing the stability of Snail, Twist, and ZEB1, and thus, it facilitates mesothelial clearance and metastasis (Kielbik et al., 2024). Lactate can promote EMT through pH-independent transforming growth factor-β (TGF-β) signaling; in OC, TGF-β is abundant in ascites and drives EMT, spheroid formation, and peritoneal dissemination (Rafehi et al., 2016; Kumari et al., 2021). A low pH inhibits the activity of weakly basic anticancer drugs, such as cisplatin, by restricting their diffusion; therefore, OC cells have acquired drug resistance through V-ATPase-mediated pH dysregulation (Kulshrestha et al., 2019). Hypoxia and acidity together select for stem-like cells with intrinsic chemoresistance; hypoxia activates TGF-β signaling to promote HIF-2α^+^ cancer stem-cell-mediated chemoresistance in OC (Zhang X. et al., 2025).

### 3.5 Metabolic symbiosis and lactate shuttling in cancer cells and CAFs

CAFs are relatively numerous in the OC microenvironment. Tumor-derived TGF-β promotes aerobic glycolysis in CAFs by upregulating glycolytic enzymes and MCT. According to the “reverse Warburg effect” model in OC, stromal CAFs perform aerobic glycolysis to produce lactate, which is exported and then consumed by neighboring cancer cells for OXPHOS (Suh et al., 2014). CAFs are rich in MCT4 and thus excrete lactate. Hypoxia and TGF-β1 cooperate to promote autophagy in CAFs and enhance aerobic glycolysis and MCT4 expression (Jena et al., 2022). Neighboring OC cells import this lactate *via* MCT1 and use it as a substrate for the TCA cycle, retaining glucose for biosynthetic pathways. OC tissue has increased mRNA levels of both MCT1 and MCT4 compared to that in normal ovarian tissue, and higher expression of MCT4 is associated with a shorter patient survival. Knockdown of MCT4 also inhibits the invasion and migration of SKOV3 cells (Chatterjee et al., 2023).

### 3.6 Therapeutic rationale for targeting glycolysis to normalize the TME

Glycolysis in OC supports the metabolism of cancer cells and simultaneously promotes an immunosuppressive TME. Therefore, the inhibition of glycolysis can be divided into two types: direct cytotoxicity to cancer cells and activation of anti-cancer immunity. A large number of studies have also found this. OC inhibits aerobic glycolysis and lactate metabolism selectively to enhance the response of cancer cells to immunotherapy (Jin Z. et al., 2025). Disruption of lactate shuttling by MCT4 represents a promising therapeutic approach, as MCT4 is a promising therapeutic direction and because MCT4 expression in OC positively correlates with gene families involved in immune modulation (Chatterjee et al., 2023). Mechanistically, LDHB regulates H3K18 lactylation on the programmed death-ligand 1 (PD-L1) promoter, directly driving PD-L1 expression and immune evasion; conversely, LDHB knockdown enhances T-cell-mediated killing, increases IFN-γ and TNF-α production, and elevates granzyme B and perforin levels (Hu et al., 2025). In addition, lactate secretion by Myc-overexpressing OC cells promotes M2-like macrophage polarization and impairs CD8^+^ T-cell function (Liu X. et al., 2024). Natural compounds that downregulate HIF-1α, HK2, LDHA, and MCT4 may reduce lactate production and alleviate TME immunosuppression. For instance, tanshinone I downregulates HK2 and LDHA expression, decreases H3K18la levels, and alleviates the immunosuppressive TME in OC (Jin B. et al., 2025).

Glycolysis supports malignant proliferation and drives immunosuppression in OC. Targeting this pathway is, therefore, a rational therapeutic strategy. Natural compounds that inhibit glycolysis may suppress tumor growth. They may also attenuate lactate-mediated immune evasion within the TME. Developing such agents addresses an unmet clinical need. These compounds could simultaneously impair cancer-cell metabolism and restore antitumor immunity by improving the TME.

## 4 Methods

To study the mechanisms through which natural compounds suppress the glycolytic pathway in OC, the PubMed, Embase, Web of Science, Science Direct, SpringerLink, Wiley, and CNKI databases were searched for articles on the chosen topic from January 2015 to November 2025. The literature included in this review encompasses three primary themes and related free-text terms: (1) “ovarian cancer” and its synonyms, including “ovarian tumor” and “ovary;” (2) “glycolysis” and related terms, including “Warburg effect,” “metabolic reprogramming,” and “Embden–Meyerhof pathway.” Articles containing terms such as “GLUT,” “HK2,” and “LDH” were also included to broaden the search scope. (3) “Natural compounds” and related terms such as “herbal medicines.” After repeated screening and evaluation based on titles and abstracts, 33 relevant natural compounds (52 relevant publications) were ultimately selected. Notably, most included studies involved *in vivo*, *in vitro*, or preclinical research, with human studies being relatively scarce.

## 5 Natural compounds targeting the glycolytic pathway in OC

**FIGURE 3:**
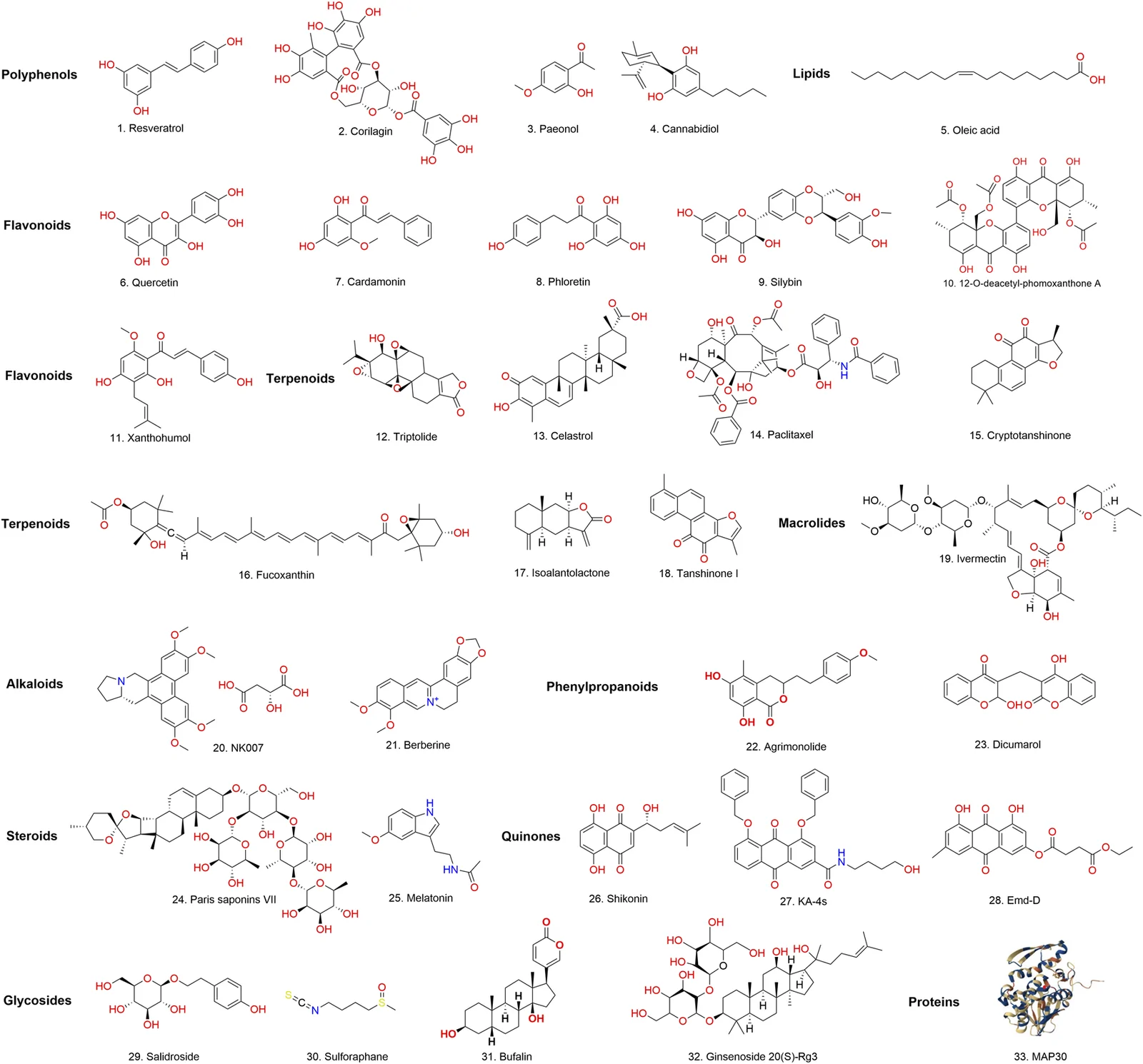
Structural formulae of the natural compounds. The MAP30 image is taken from the RCSB PDB (RCSB.org) of PDB ID 1BNA (H.R. Drew, R.M. Wing, T. Takano, C. Broka, S. Tanaka, K. Itakura, R.E. Dickerson, Structure of a B-DNA dodecamer: conformation and dynamics (1981) Proc. Natl.Acad.Sci.USA 78: 2179–2183) and the article (Zhao Y, Lu L, Chen X, Yin Q) Natural compounds targeting ferroptosis in ovarian cancer: research progress and application potential (Pharmacol Res. 2025 May; 215:107729.).

**FIGURE 4:**
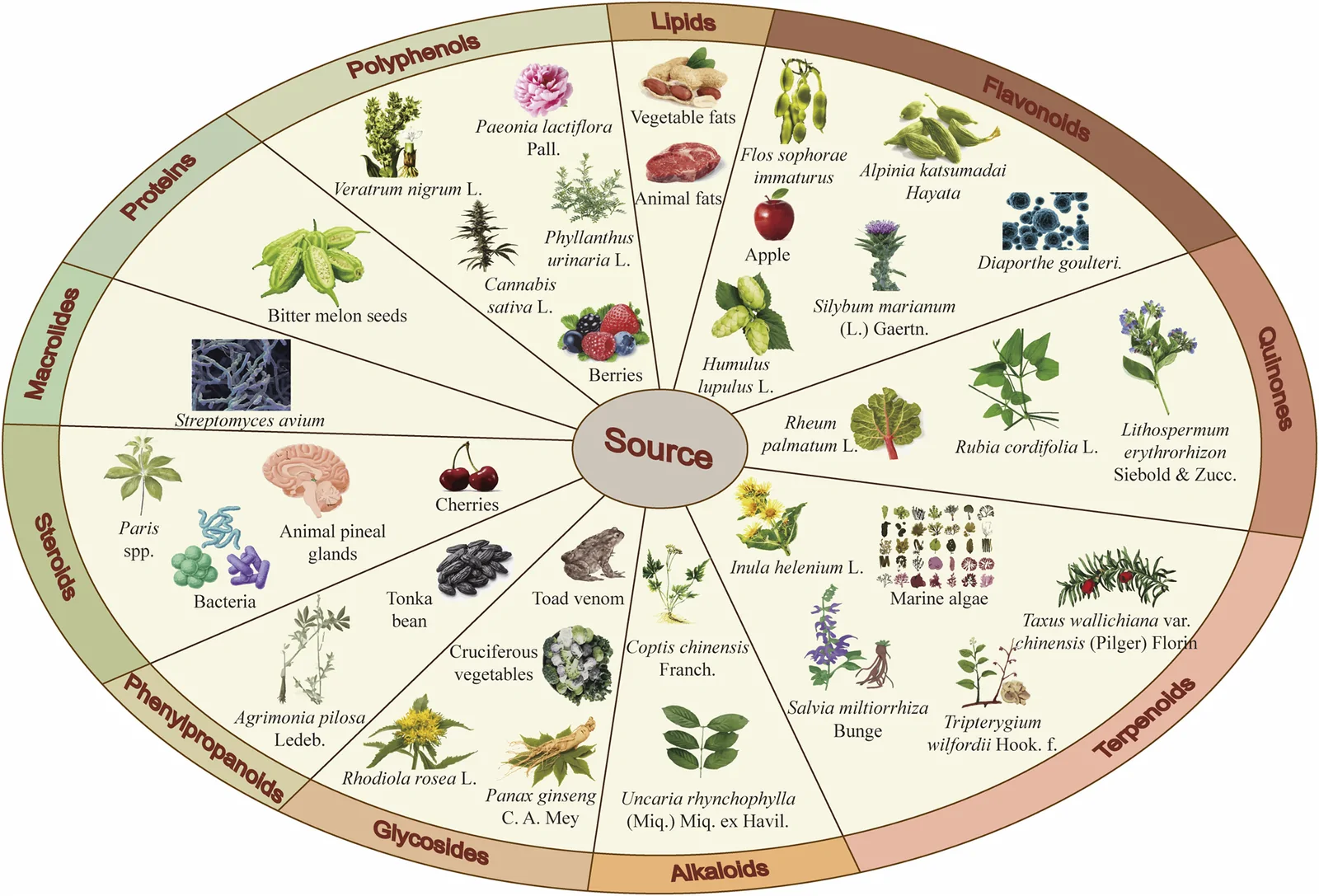
Sources of natural compounds. Classification of natural bioactive compounds with pharmacological potential based on chemical structural categories. The inner circle summarizes the origin of bioactive substances (plants, microorganisms, or animals). The middle ring divides compounds into 11 major chemical classes.

Several fundamental studies have identified a wide array of natural compounds that exhibit diverse anticancer activities, are capable of mitigating adverse reactions during cancer treatment, or can attenuate drug resistance. By screening for agents that target OC through the inhibition of glycolysis-related enzymes or genes, researchers have identified numerous plant- and microorganism-derived compounds whose anticancer effects were subsequently verified in both *in vitro* and *in vivo* models. These compounds are categorized based on their molecular structures in Figures 3, 4 and Tables 1–3.

**TABLE 1:** Natural compounds targeting glycolysis for OC therapy (↑ increase, ↓ decrease).

Compound	Source	*In vitro*	Dosage	*In vivo*	Dosage	Mechanism	Effects
Resveratrol (Liu et al., 2018)	*Veratrum nigrum* L., Berries	A2780, SKOV3, and IOSE-80	0 μM–800 μM (24 h–72 h)	SKOV3 cells xenograft model	100 mg/kg/day, 18 days	↓glucose and lactic acid ↑AMPK/mTOR	Inhibits proliferation, migration, and invasion and induces apoptosis
Corilagin (Jia et al., 2017)	*Phyllanthus urinaria* L.	SKOV3, OVCAR5, OC316, Hey, HOPM, and HOPM^−Snail^	20 μM–40 μM (24, 48, and 72 h)	—	—	↓Snail, CD44, STAT3, PKM2, and phospho-ERK	Enhances the sensitivity of ovarian cancer cells to chemotherapy
Paeonol (Yang et al., 2025)	*Paeonia lactiflora* Pall.	SKOV3, SKOV3/DDP, and IOSE80	500 μM (24, 48, and 72 h)	SKOV3/DDP cells xenograft model	40 mg/kg, 28 days, administer the next day	↓ACSS2, HK2, PKM1, lactic acid, acetyl-CoA, ECAR, and D-glucose ↑LDHA, PDHA1, SIRT1, ATG5, ATG2, and BOXPHOS	Suppresses proliferation, migration. and invasion; induces apoptosis; and reduces their chemotherapy resistance
Cannabidiol (Ma et al., 2023)	*Cannabis sativa* L.	SKOV3 and CAOV3	20 and 40 μM (24 and 48 h)	CAOV3 cells xenograft model	40 mg/kg/day 12 days	↓OCR, ATP, and ECAR, PI3K/AKT/mTOR ↑ROS, caspase-3, and caspase-9	Induces ovarian cancer cell-cycle arrest and promotes cell apoptosis
Oleic acid (Kado et al., 2023)	Animal fats and vegetable fats	HNOA and RMG-1	10 and 30 μM (24 and 48 h)	—	—	↑PPARα, BRD4-L-MYC-GLUT, ATP, and lactic acid	Promotes glycolysis and cell proliferation
Quercetin (Xintaropoulou et al., 2015)	*Flos sophorae immaturus*	OVCAR3, OVCAR5, CAOV3, and TOV112D	0 μM–300 μM (5 days)	—	—	↓lactic acid, GLUT1, HK2, PFKFB3, PDHK1, and LDH	Blocks glycolysis and increases apoptosis
Cardamonin (Shi et al., 2018)	*Alpinia katsumadai Hayata*	SKOV3	5, 10, and 20 μM (12, 24, 36, 48, and 60 h)	—	—	↓mTORC1, lactic acid, HK2, ATP, and LDH	Inhibits proliferation and enhances autophagy
Phloretin (Xintaropoulou et al., 2015)	Apple	OVCAR3, OVCAR5, CAOV3, and TOV112D	0 μM–300 μM (5 days)	—	—	↓lactic acid, GLUT1, HK2, PFKFB3, PDHK1, and LDH	Blocks glycolysis and increases apoptosis
Silybin (Wei et al., 2022)	*Silybum marianum* (L.) Gaertn.	OVCAR3 and SKOV3	50 and 100 μM (24 48 h)	OVCAR3 cells xenograft model	100 mg/kg/day, 6 weeks	↓IDH1, OCR, and ATP ↑ROS	Suppresses tumor proliferation by interfering with the redox balance
12-ODPXA (Yang C. et al., 2024)	*Diaporthe goulteri.*	IOSE80, SKOV3, and ES-2	1, 2, 4, 8, 16, and 32 μM (24, 48, and 72 h)	SKOV3 cells zebrafish xenograft model	0.1 μg/mL 1 μg/mL 10 μg/mL	↓PDK4, glucose, lactic acid, and ATP	Inhibits PDK4 activity, reduces cellular glycolysis, and consequently hinders proliferation, metastasis, and invasion
Xanthohumol (He et al., 2024)	*Humulus lupulus* L.	SKOV3 and A2780	1, 2, and 4 μM (24, 48, and 72 h)	SKOV3, A2780 cells xenograft model	3 mg/kg every 2 days 10 mg/kg every 2 days 28 days	↓Skp2/AKT/HK2 ↑caspase 3	Inhibits aerobic glycolysis and cell viability of ovarian cancer cells
Shikonin (Zhou D. et al., 2022)	*Lithospermum erythrorhizon* Siebold and Zucc.	A2780, SKOV3, and OVCAR3	4, 6, and 8 μM (2 h)	A2780 cells xenograft model	2 mg/kg every 2 days 12 days	↓PKM2	Reduces cell growth, colony formation, and migration and induces cell apoptosis.
KA-4s (Zhao et al., 2024a)	*Rubia cordifolia* L., *Rheum palmatum* L.	SKOV3 and SKOV3/DDP	0 μM–20 μM (24 and 48 h)	SKOV3, SKOV3/DDP cells xenograft model	3 and 6 mg/kg/day, 40 days	↓GLUT1, GLUT4, PKM2, ATP, OCR, and glycoPER ↑AMPK	Promotes the apoptosis of drug-resistant ovarian cancer cells and inhibits cell migration and invasion.
Emd-D (Zhao et al., 2025)	*Rheum palmatum* L.	OVHM and SKOV3	10, 30, and 100 μM (12 and 24 h)	OVHM, SKOV3 cells xenograft model	20, 60, and 180 mg/kg/day 21 days	↓PFKFB4, lactate, pyruvate, GLUT1, and HK2	Induces apoptosis.
Triptolide (Wang et al., 2018)	*Tripterygium wilfordii* Hook. f.	A2780 and A2780/CP70	100 nM (24 h)	—	—	↓HK2	Promotes cisplatin-induced cell apoptosis and decreases the viability and survival fraction.
Celastrol (Wang et al., 2022)	*Tripterygium wilfordii* Hook. f.	A2780	100, 500, and 1,000 nM (24 and 96 h)	—	—	↓HIF-1α and MYC	Regulates cell proliferation, DNA repair and replication, apoptotic processes, and inflammatory responses.
Fucoxanthin (Zhang et al., 2023)	Marine algae	A2780 and OVCAR3	25, 50, 75, and 100 μM (48 h)	—	—	↓F-FDG, ATP, ECAR, LDHA, HK2, PDK1, and STAT3/c-Myc	Suppresses cell proliferation and metastasis.
Isoalantolact-one (Chun, 2023)	*Inula helenium* L., *Inula racemosa* Hook.f.	A2780^sen^, PEO1^sen^, A2780^cisR^, PEO1^cisR^, SNU-8^sen^, and SNU-8^cisR^	5, 10, and 15 μM (24 and 48 h)	A2780^sen^ and A2780^cisR^ cells xenograft model	20 mg/kg/day 17 days	↓lactic acid, ATP, LDHA, PFKL, CS, PDHA1, HK2, and GLUT1 ↑PARP1, caspase-3, and caspase-8	Reduces cisplatin resistance.
Paclitaxel (Lin Y. et al., 2024)	*Taxus wallichiana* var. *chinensis* (Pilger) Florin	ES2, ES2TR120, and OSE2a	0 nM–250 nM (24, 48, 72, and 96 h)	—	—	↑HK2	Restores sensitivity to paclitaxel
Cryptotanshi-none (Wang T. et al., 2024)	*Salvia miltiorrhiza* Bunge	A2780, CAOV3, and IOSE80	0 μM–20 μM (0 h–72 h), 1, 2, and 5 μM (12–14 days)	A2780 cells xenograft model	10 mg/kg every 3 days 24 days	↓glucose, lactic acid, LDHA, HK2, GLUT1, PKM2, Complex-1, ATP, and OXPHOS ↑AMPK	Inhibits growth and induces apoptosis
Tanshinone I (Jin B. et al., 2025)	*Salvia miltiorrhiza* Bunge	A2780 and ID-8	0.5, 5, and 50 μg/mL (12, 24, and 36, 48 h)	A2780 cells xenograft model	10 mg/kg every 3 days 30 days	↓HK2, PFK, ENO2, LDHA, H3K18la, and lactic acid	Inhibits cancer cell growth
NK007 (Li et al., 2019)	*Uncaria rhynchophylla* (Miq.) Miq. ex Havil.	A2780, A780^Taxol^, 2774-C10, OVCAR3, and H08910	0 nM–2,000 nM (24, 48, and 72 h)	—	—	↓HK2, OCR, and ECAR	Overcomes paclitaxel resistance through p38MAPK activation and HK2 degradation
Berberine (Yan et al., 2024)	*Coptis chinensis* Franch.	SKOV3 and HEY	1 μM–128 μM (24, 48, and 72 h)	HEY^LINC01123^ and HEY^siLINCO1123^ cells xenograft model	10 and 20 mg/kg, 4 weeks	↓LINC00123/P65/MAPK10, glucose, lactic acid, OCR, ECAR, NADPH, ATP	Inhibits ovarian cancer proliferation and metastasis by modulating autophagy and glycolysis levels
Sulforaphane (Pastorek et al., 2015)	Cruciferous vegetables	A2780, A2780/ADR, and A2780/CP	2.5, 5, and 10 μM (24 h–48 h)	—	—	↓HIF-1α, CA IX, and AP-1 ↑p53, ARE, IRF-1, Pax-6, and XRE	Reduces migration and overcomes the chemoresistance of ovarian carcinoma cells
Salidroside (Yu and Feng, 2024)	*Rhodiola rosea* L.	IOSE80, CAOV3, and SKOV3	0, 50, 100, 200, 400, and 800 μM (24 h)	SKOV3 cells xenograft model	80 mg/kg every 2 days 27 days	↓STAT3/c-Myc, glucose, lactic acid, ATP, ECAR, GLUT1, HK2, and LDHA	Reduces OC cell viability, migration and invasiveness, induces apoptosis, and inhibits cellular EMT by inhibiting glycolytic processes
Ginsenoside 20(S)-Rg3 Li et al. (2015)	*Panax ginseng* C. A. Mey.	SKOV3 and 3AO	160 μg/mL (SKOV3), 80 μg/mL (3AO), (24 and 48 h)	—	—	↓STAT3/HK2, PKM, GLUT1, LDH, and PFK	Inhibits the Warburg effect and tumor growth
Bufalin (Li H. et al., 2018)	Toad venom	A2780 and Hey	0 nM–320 nM (24, 48, and 72 h)	A2780 cells xenograft model	10 mg/kg every 2 days	↓ITGB2/FAK, glucose, lactic acid, GLUT4, LDHB, and HK2	Inhibits cellular glycolysis-induced cell growth and proliferation
Dicumarol (Zhang et al., 2017)	Tonka bean	SKOV3 and A2780	100 and 200 μM (24 h)	SKOV3 cells xenograft model	30 and 50 mg/kg every 2 days 12 days	↓PDK1, OCR, and ECAR ↑ROS	Induces apoptosis and reduces cell viability
Agrimonolide (Yang C. et al., 2024)	*Agrimonia pilosa* Ledeb.	SKOV3 and A2780	10, 20, and 40 μM (24, 48, and 72 h)	-	-	↓glucose, lactic acid, HIF-1α, HK2, and LDHA	Restrains glycolysis in ovarian cancer cells and inhibits tumor growth
*Paris* saponins VII (Wang M. et al., 2024)	*Paris* spp.	HEY and SKOV3	2 and 4 μM (24, 48, 72, and 96 h)	SKOV3^PARPi−R^, HEY^PARPi−R^ cells xenograft model	30 days	↓glucose, lactic acid, ATP, OCR/ECAR, and NADPH	Hinders glycolysis and angiogenesis in PARPi-resistant ovarian cancer cells by binding and stabilizing the expression of RORα
Melatonin (Silveira et al., 2024)	Animal pineal glands, cherries, and bacteria	SKOV3 and CAISMV24	2.0, 3.4, 5.0, and 7.0 μM (24 h)	—	—	↓HIF-1α, PDH, PFK-1, LDH, CS, GS, CREB, JNK, NF-kB, p-38, ERK1/2, AKT, P70S6K, and STAT	Reverses Warburg-type metabolism, reduces glutaminolysis, and inhibits tumor growth
Ivermectin (Li et al., 2024)	*Streptomyces aviumitilis*	TOV-21G	5 and 10 μM (12 and 24 h)	TOV-21G cells xenograft model	5 and 10 mg/kg/day, 15 days	↓PFK, PKM, DLD, DLAT, PDH, LDH, ENO1, ADH5, and GPI	Inhibits cell proliferation
MAP30 (Chan et al., 2020)	Bitter melon seeds	A2780cp, HEY, ES2, HEYA8, HEK293, OVCA433, SKOV3, HOSE11-12, HOSE96-9–1, and AMPKγ2-HEK293	5, 10, 20, 30, and 40 μg/mL (15, 24, 48, and 72 h)	ES2 cells xenograft model	62.5, 125, 250, and 500 μg/kg every 2 days 40 days	↓GLUT-1/3, AKT/ERK/FOXM1/mTOR, glucose, lactic acid, LDH, and HK2 ↑AMPK and ROS	Inhibits migration, invasion, and proliferation

**TABLE 2:** Natural compounds remodel the OC tumor microenvironment through glycolysis (↑ increase, ↓ decrease).

Compound	Key glycolytic targets	TME-specific effects
Resveratrol (Chen et al., 2022a)	↓Extracellular lactic acid	Impairs the suppressive function of regulatory T cells and increases CD4^+^ and CD8^+^ T-cell infiltration into the TME
Corilagin (Jia et al., 2017)	↓LDHA and ECAR	Blocks TGF-β-induced Snail upregulation and suppresses the EMT
Paeonol (Yang et al., 2025)	↓Extracellular lactic acid	Ameliorates the TME and contributes to the reversal of chemoresistance
Oleic acid (Kado et al., 2023)	↑Extracellular lactic acid	Contributes to an immunosuppressive TME
Quercetin (Xintaropoulou et al., 2015)	↓Extracellular lactic acid	Ameliorates the TME and leads to an increase in apoptosis
Cardamonin (Shi et al., 2018)	↓Extracellular lactic acid	Ameliorates the acidic TME
Phloretin (Xintaropoulou et al., 2015)	↓Extracellular lactic acid	Ameliorates the TME and results in apoptosis
12-ODPXA (Yang C. et al., 2024)	↓Extracellular lactic acid	Ameliorates the TME
Xanthohumol (Liu Y. et al., 2019)	↓Extracellular lactic acid	Ameliorates the acidic TME and limits metastasis
Triptolide (Ding et al., 2025)	↓Extracellular lactic acid	Attenuates exosomal ITGA5-induced migration of human peritoneal mesothelial cells and mitigates lactate-driven metabolic reprogramming
Isoalantolactone (Chun, 2023)	↓Extracellular lactic acid	Ameliorates the acidic TME
Paclitaxel (Feng et al., 2022; Wu et al., 2022)	↓Extracellular lactic acid	Ameliorates the acidic TME
Cryptotanshinone (Yang Y. et al., 2018; Wang T. et al., 2024)	↓Extracellular lactic acid	Ameliorates the TME
Tanshinone I (Jin Z. et al., 2025)	↓Extracellular lactic acid, H3K18la	Increases intra-tumoral CD4^+^ and CD8^+^ T cells, decreases Tregs, reduces M2-like tumor-associated macrophages, and contributes to an immunosuppressive TME
Berberine (Zhang Z. et al., 2025)	↓Extracellular lactic acid	Increases intra-tumoral CD4^+^ and CD8^+^ T-cell infiltration, reduces PD-L1 and NF-κB expression, and promotes the proliferation of tumor-infiltrating immune cells, thereby ameliorates the immunosuppressive TME and suppresses OC metastasis
Sulforaphane (Pastorek et al., 2015)	↓HIF-1α and CA IX	Ameliorates the acidic TME, inhibits cell migration, and reverses chemoresistance
Salidroside (Yu and Feng, 2024)	↓Extracellular lactic acid	Suppresses the EMT
Ginsenoside 20(S)-Rg3 Li et al. (2015), Zheng et al. (2018), Lu et al. (2019), 2020	↓Extracellular lactic acid	Ameliorates the acidic TME
Bufalin (Li X. et al., 2018)	↓Extracellular lactic acid	Ameliorates the acidic TME
Dicumarol (Zhang et al., 2017)	↓Extracellular lactic acid	Decreases the MMP and ameliorates the acidic TME
Agrimonolide (Yang C. et al., 2024)	↓Extracellular lactic acid	Ameliorates the acidic TME
*Paris* saponins VII (Wu Z. et al., 2023)	↓Extracellular lactic acid	Ameliorates the acidic TME
Melatonin (Silveira et al., 2024)	↓Extracellular lactic acid and glutamine levels	Limits lactate efflux and ameliorates the TME acidification
Ivermectin (Li Y. et al., 2020)	↓Extracellular lactic acid	Downregulates the MCT1 and MCT4, limits lactate efflux, ameliorates the TME acidosis, and relieves associated immunosuppression
MAP30 (Chan et al., 2020)	↓Extracellular lactic acid	Reduces total fatty acid levels in malignant ascites and reversal of OCM-induced lipid droplet accumulation, remodels the nutrient landscape of the peritoneal TME, and contributes to the suppression of tumor dissemination *in vivo*

**TABLE 3:** A comprehensive summary of the molecular mechanisms and evidence tier by natural compounds target glycolysis in ovarian cancer. (↑ increase, ↓ decrease; evidence tier: I = *in vitro* only; II = *in vitro* + *in vivo*; III = includes clinical/human data. All compounds listed are tier I or II unless otherwise noted).

Compound	GLUT	HK2	PFK/PFKFB	PKM	LDH	PDK	Lactate	STAT3/HIF-1α/c-Myc	Main signaling pathway(s)	Evidence tier
Resveratrol (Liu et al., 2018)	↓ GLUT1	—	—	↓ PKM2	—	—	↓	—	↑AMPK/mTOR	I I
Corilagin (Jia et al., 2017)	—	—	—	—	↓ LDHA	—	—	↓ STAT3/Snail	↓STAT3/Snail	I I
Paeonol (Yang et al., 2025)	—	↓	—	↓ PKM1	—	—	↓	​	↓ACSS2	I I
Cannabidiol (Ma et al., 2023)	—	—	—	—	—	—	↓	—	↓PI3K/AKT/mTOR	I I
Oleic acid (Kado et al., 2023)	↑ GLUT1/3/4	—	—	—	—	—	↑	↑ c—Myc	↑PPARα/BRD4-L-MYC	I
Quercetin (Xintaropoulou et al., 2015)	↓ GLUT1	↓	↓ PFKFB3	—	↓ LDH	↓ PDK1	↓	—	Multi-enzyme inhibition	I
Cardamonin (Shi et al., 2018)	—	↓	—	—	↓ LDH	—	↓	—	↑AMPK/↓mTORC1	I
Phloretin (Xintaropoulou et al., 2015)	↓ GLUT1	↓	↓ PFKFB3	—	↓ LDH	↓ PDK1	↓	—	Multi-enzyme inhibition	I
Silybin (Wei et al., 2022)	↓ GLUT4	—	—	—	—	—	—	—	↓IDH1	I I
12-ODPXA (Yang G. et al., 2024)	—	—	—	—	—	↓ PDK4	↓	—	PDK4 degradation	I I
Xanthohumol (He et al., 2024)	—	↓	—	—	—	—	↓	—	↓Skp2/AKT	I I
Shikonin (Zhou D. et al., 2022)	—	—	—	↓ PKM2	—	—	—	—	Direct PKM2 inhibition	I I
KA-4s (Zhao et al., 2024b)	↓ GLUT1/4	—	—	↓ PKM2	—	—	—	—	↑AMPK	I I
Emd-D (Zhao et al., 2025)	↓ GLUT1	↓	↓ PFKFB4	—	—	—	↓	—	↓SRC-3/mTORC1	I I
Triptolide (Wang et al., 2018)	—	↓	—	—	—	—	↓	—	↓ITGA5	I I
Celastrol (Wang et al., 2022)	—	—	—	—	—	—	—	↓ HIF-1α/Myc	↓ HIF-1α/Myc	I
Fucoxanthin (Zhang et al., 2023)	—	↓	—	—	↓ LDHA	↓ PDK1	—	↓ STAT3/c-Myc	↓STAT3/c-Myc	I
Isoalantolactone (Chun, 2023)	↓ GLUT1	↓	↓ PFKL	—	↓ LDHA	—	↓	​	Modulates MAPK/AKT	I I
Paclitaxel (Lin T.-Y. et al., 2024)	​	↑	↓ PFKFB2	—	—	—	—	—	Multiple resistance pathways	I I
Cryptotanshinone (Wang T. et al., 2024)	↓ GLUT1	↓	—	↓ PKM2	↓ LDHA	—	↓	↓ STAT3/SIRT3	↓STAT3/SIRT3/HIF-1α	I I
Tanshinone I (Jin B. et al., 2025)	—	↓	↓ PFK	—	↓ LDHA	—	—	-	↓PDGFRβ/YTHDF2	I I
NK007 (Li et al., 2019)	—	↓	—	—	—	—	—	—	p38MAPK activation	I
Berberine (Yan et al., 2024)	↓ GLUT4	↓	—	—	↓ LDHB	—	↓	—	↓LINC01123/P65	I I
Sulforaphane (Pastorek et al., 2015)	↓ GLUT1	—	—	—	—	—	—	↓ HIF-1α	↓HIF-1α/CA IX	I
Salidroside (Yu and Feng, 2024)	↓ GLUT1	↓	—	—	↓ LDHA	—	↓	↓ STAT3/c-Myc	↓STAT3/c-Myc	I I
20(S)-Ginsenoside Rh2 Li et al. (2015)	↓ GLUT1	↓	↓ PFK	↓ PKM2	↓ LDH	—	↓	↓ STAT3/HIF-1α	↓STAT3/HK2; ↑miRNAs	I I
Bufalin (Li X. et al., 2018)	↓ GLUT4	↓	—	—	↓ LDHB	—	↓	—	↓ITGB2/FAK	I I
Dicumarol (Zhang et al., 2017)	—	—	—	—	—	↓ PDK1	↓	—	Shifts to OXPHOS	I I
Agrimonolide (Yang C. et al., 2024)	—	↓	—	—	↓ LDHA	—	↓	↓ HIF-1α	↓ HIF-1α	I
Paris saponins VII (Wang T. et al., 2024)	↓ Glucose uptake	—	—	—	—	—	↓	—	↑RORα/RORC	I I
Melatonin (Silveira et al., 2024)	—	—	↓ PFK-1	—	↓ LDH	—	↓	↓ HIF-1α	Multi-pathway modulation	I
Ivermectin (Li et al., 2024)	—	—	↓ PFKP	↓ PKM	↓ LDH	—	—	—	Multi-enzyme inhibition	I I
MAP30 (Chan et al., 2020)	↓ GLUT1/3	↓	—	—	↓ LDH	—	↓	—	↑AMPK; ↓AKT/mTOR	I I

The compounds identified to date fall into several chemical classes, including polyphenols, lipids, flavonoids, quinones, terpenoids, alkaloids, glycosides, phenylpropanoids, steroids, macrolides, and proteins. In the following subsections, we systematically present their sources, experimental models, mechanisms, and effects on glycolysis and the TME. For each compound, we explicitly indicate whether the evidence is derived from *in vitro* studies only or it has been validated in *in vivo* models; clinical data, where available, are highlighted separately.

### 5.1 Polyphenols

#### 5.1.1 Resveratrol (RV)

RV is a natural phytoalexin that several plants produce, such as rhubarb, berries, grapes, and *Polygonum cuspidatum*. This compound exhibits a broad spectrum of biological activities, including antioxidant, anti-inflammatory, and immune-boosting effects. Beyond these properties, RV effectively inhibits glycolytic metabolism in tumor cells (Ren et al., 2021; Brockmueller et al., 2024). Incubating A2780 and SKOV3 cells with 200 μM RV reduced their cell viability by approximately 37.67% and 70.25%, respectively, and inhibited glucose uptake effectively. In addition, the lactate concentration of the RV-treated cells was also lower than that in the control group (Liu et al., 2018). RV treatment in xenograft C57BL/6 mouse models reduced lactate concentration in the TME and downregulated the expression of PKM2 and GLUT1 in tumor tissues. The reduction in lactate impaired the suppressive function of regulatory T cells and expanded the infiltration of CD4^+^ and CD8^+^ T cells in the TME (Chen Y. et al., 2022). In IL-6-enriched inflammatory conditions that mimic the TME, RV inhibited GLUT1 membrane translocation and glucose uptake, restored autophagic flux, and thus suppressed OC-cell migration (Vidoni et al., 2023). Thus, RV inhibits the development of OC *via* glycolytic inhibition, TME immunomodulation, and autophagic restoration.

#### 5.1.2 Corilagin

Corilagin is an epigallocatechin gallate tannin that occurs in longan, euryale seed, rambutan, amla, and olive, as well as in medicinal plants such as *Phyllanthus urinaria*, *Impatiens grandiflorum*, *Terminalia chebula*, white clover, and *Phyllanthus mauritiana* (Li H. et al., 2018). It exhibits multiple pharmacological activities, including antitumor, antioxidant, hepatoprotective, and anti-inflammatory effects (Attar et al., 2017; Luo et al., 2022). Analysis of the extracellular acidification rate (ECAR) in Skip, HEY, HOPM, and HOPM-Snail cells treated with 25 μM corilagin confirmed that the compound inhibits glycolysis by downregulating the expression of cell adhesion molecule 44 (CD44) and signal transducer and activator of transcription 3 (STAT3) and suppressing LDHA and ENO1. Notably, HOPM-Snail cells displayed the greatest extent of glycolytic suppression. Coriagin also downregulated Snail expression and blocked TGF-β-induced Snail upregulation in OC cells. Snail is a central transcriptional regulator of EMT and is induced by TME-derived cytokines including TGF-β (Jia et al., 2017). These findings indicate that corilagin sensitizes cancer epithelial cells to paclitaxel and carboplatin treatment by inhibiting the Snail-glycolytic pathway.

#### 5.1.3 Paeonol (Pae)

Paeonol (Pae), a natural phenolic monomer and an early synthetic isolate of peony root bark, displays pharmacological properties, including anti-inflammatory, neuroprotective, and antitumor activities, along with effects against cardiovascular disease (Zhao et al., 2014; Zhang L. et al., 2019; Liu et al., 2025). The treatment of OC cell lines with 500 μM Pae led to a marked downregulation of glycolysis-related genes, including those encoding major enzymes such as HK2 and PKM-1, alongside a decrease in glucose uptake. The *in vivo* data were consistent with these *in vitro* findings. Furthermore, analysis of the ECAR in SKOV3/DDP cells showed that Pae reduced glycolytic flux while simultaneously increasing the oxygen consumption rate (OCR). In SKOV3/DDP cells, Pae also decreased the lactate levels, an effect partially reversed by glucose supplementation, which may ameliorate the TME and thereby contribute to the reversal of chemoresistance (Yang et al., 2025).

#### 5.1.4 Cannabidiol (CBD)

Cannabidiol (CBD), a pure natural compound extracted from the cannabis plant, exhibits anti-anxiety, analgesic, anti-epileptic, anti-inflammatory, and antitumor effects. It has demonstrated antitumor activity against multiple cancer types (Campos et al., 2016; Yan et al., 2023). SKOV3 and CAOV3 cells treated with 15, 20, 40, and 60 μM CBD exhibited significantly reduced aerobic glycolysis rates and mitochondrial respiration. These effects were confirmed through analysis of the ECAR and OCR. *In vivo* and *in vitro* experiments further demonstrated that CBD inhibits OC cell growth by disrupting leukocyte-associated immunoglobulin-like receptor 1 (LAIR1)-mediated interference with mitochondrial bioenergetics and the PI3K/AKT/mTOR signaling pathway. However, these mechanisms were limited to promoting cell-cycle arrest and apoptosis (Ma et al., 2023).

### 5.2 Lipids

#### 5.2.1 Oleic acid (OA)

The source of oleic acid (OA) is primarily natural, existing mainly as glycerides in various animal and plant fats. It supports cardiovascular health, improves the regulation of blood glucose levels, and boosts immunity (Santa-María et al., 2023). Conversely, in the context of malignancy, OA promotes cancer-cell proliferation (Yang P. et al., 2018; Sun Y. et al., 2024). Following the treatment of HNOA cells with 10 and 30 μM OA, reverse transcription polymerase chain reaction (RT-PCR) analysis confirmed that OA treatment upregulated the expression of GLUT1, GLUT3, and GLUT4. In a reverse validation experiment, PFK158, an inhibitor of a rate-limiting glycolytic enzyme, suppressed OA-induced cell proliferation. OA also increased the level of extracellular lactate and may have promoted an immunosuppressive TME (Kado et al., 2023). OA can help the HNOA cells live by increasing glucose consumption (Kuwata et al., 2013). OA extends the survival rate of serum-starved HNOA cells by increasing glucose consumption and, thus, increasing the production of both ATP and lactate.

### 5.3 Flavonoids

#### 5.3.1 Quercetin (QR)

Quercetin (QR) is a GLUT1-inhibitor that occurs in high amounts in many vegetables, fruits, medicinal plants, and grains. The first natural sources include *Sophora japonica* flowers, onions, apple peels, and tea leaves (Deepika and Maurya, 2022; Baqer et al., 2024). It exhibits several biological properties, including tumor-inhibiting, antioxidant, antibacterial, and anti-inflammatory effects, and it has also been reported to modulate blood pressure, lipid levels, and immune function (Vafadar et al., 2020; Hosseini et al., 2021; Ma et al., 2022). The treatment of OVCAR5, TOV112D, OVCAR3, and CAOV3 cell lines with 0.6 μM–300 μM QR led to the inhibition of five key molecules in the glycolytic pathway: GLUT1, HK2, PFKFB3, pyruvate dehydrogenase kinase 1 (PDK1), and lactate dehydrogenase (LDH). This widespread inhibition blocked glycolysis, leading to increased glucose concentrations outside OC cells and reduced extracellular lactate production, thereby ameliorating the TME and ultimately leading to an increase in apoptosis (Xintaropoulou et al., 2015).

#### 5.3.2 Cardamonin (CDN)

Cardamonin (CDN) is a natural chalcone derived from cardamom, which is obtained from Chinese medicinal herbs, including black and white cardamom (Gonçalves et al., 2014). This compound exhibits antitumor, antioxidant, anti-inflammatory, and hepatoprotective activities (Karimi-Sales et al., 2018; Nawaz et al., 2020; Xu et al., 2020; Al-Sultany et al., 2023). The treatment of SKOV3 cells with 5, 10, or 20 μM CDN significantly inhibited extracellular lactate levels in a dose- and time-dependent manner, with maximal inhibition observed after 48 h, while also reducing HK2 and LDH activity. Similarly, the intracellular ATP content decreased in a dose-dependent manner at the 48-h mark. Adenosine monophosphate-activated protein kinase (AMPK) functions as a cellular energy sensor and is typically activated in response to decreases in ATP levels. In SKOV3 cells, treatment with 20 μM CDN significantly increased the expression of HK2 and AMPK phosphorylation levels. Furthermore, CDN suppressed the phosphorylation of mTOR and its downstream target, ribosomal protein S6 kinase beta-1 (S6K1), which is consistent with the observed glycolytic suppression. These findings collectively indicate that the CDN-induced inhibition of cell proliferation correlates with the downregulation of mTOR-mediated glycolysis (Shi et al., 2018). Furthermore, in MDA-MB-231 breast cancer cells, CDN inhibited glucose utilization and lactate production by suppressing HIF-1α-mediated cellular metabolism, leading to the inhibition of cell growth both *in vitro* and *in vivo* (Jin et al., 2019).

#### 5.3.3 Phloretin

Phloretin is a natural bioactive compound widely found in the juices of apples and pears and in a variety of vegetables. It acts as a GLUT1 inhibitor and possesses several biological properties, including antioxidant, antitumor, anti-inflammatory, and anti-diabetic effects (Nakhate et al., 2022; Tuli et al., 2022; Shelke et al., 2024; Zeng et al., 2024). The treatment of OC with 0.6 μM–300 μM phloretin led to a dose-dependent suppression of cell proliferation. Additionally, phloretin inhibited the expression of GLUT1, HK2, PFKFB3, PDK-1, LDH, and extracellular lactate levels, which blocked glycolysis in tumor cells, thereby ameliorating the TME and ultimately leading to apoptosis (Xintaropoulou et al., 2015).

#### 5.3.4 Silybin (SIL)

Silybin (SIL) is a flavonoid compound isolated from the milk thistle fruit. Its pharmacological properties include antioxidant, antitumor, and hepatoprotective effects (Raina and Agarwal, 2007; Abenavoli et al., 2018; Pignatelli et al., 2019). At concentrations of 50 or 100 μM, SIL inhibited OVCAR3 cell proliferation over 24 h by increasing the production of reactive oxygen species (ROS). OCR measurements indicated that basal respiration, maximal respiration, and ATP production were all reduced. However, analysis of the ECAR showed that SIL did not significantly influence glucose utilization or lactate production in OC cells. Evidence from xenograft mouse models further demonstrated that the oral administration of SIL inhibited ovarian tumor growth. However, these mechanisms did not involve the TME (Wei et al., 2022). In another study, SIL was reported to modulate glucose uptake in adipocytes by blocking GLUT4 (Zhan et al., 2011).

#### 5.3.5 12-O-Deacetyl-phomoxanthone A (12-ODPXA)

12-O-Deacetyl-phomoxanthone A (12-ODPXA) is a flavonoid derived from the secondary metabolites of the endophytic fungus *Diaporthe goulteri.* An analog of phomoxanthone A (PXA), 12-ODPXA displays antibacterial and cytotoxic properties, alongside potent anticancer effects (Shiono et al., 2013; Zhou G. et al., 2025). In a zebrafish xenograft model, the migratory capacity of SKOV3 cells decreased in a dose-dependent fashion following exposure to 12-ODPXA. Furthermore, the incubation of SKOV3 and ES-2 cells with 0 μM–8 μM 12-ODPXA for 24 h reduced the expression of pyruvate dehydrogenase kinase 4 (PDK4). This downregulation led to diminished glucose uptake, extracellular lactate secretion, and intracellular ATP production, thereby ameliorating the TME. However, pretreatment with the proteasome inhibitor MG-132 significantly attenuated the 12-ODPXA-induced reduction in PDK4 expression, indicating that the compound promotes the proteasomal degradation of this enzyme in OC cells. Collectively, the above results indicate that 12-ODPXA inhibits cancer cells by inhibiting the Warburg effect through the downregulation of PDK4, both *in vitro* and *in vivo* (Yang G. et al., 2024).

#### 5.3.6 Xanthohumol (Xn)

Xanthohumol (Xn) is a flavonoid extract isolated from hops. It exhibits diverse biological properties, such as the ability to regulate lipid metabolism, inhibit cancer-cell proliferation, and delay neurodegenerative disease progression (Girisa et al., 2021; Gao et al., 2024; Długosz et al., 2025). S-phase kinase-associated protein 2 (Skp2) plays an essential role in OC cell growth and aerobic glycolysis. The treatment of OC cells with various concentrations of Xn (1, 2, or 4 μM) dose-dependently decreased Skp2 and HK2 expression, AKT phosphorylation, glucose consumption, and lactate production. In A2780 cell-derived xenografts, Xn treatment significantly reduced tumor volume, mass, and weight (He et al., 2024). Additionally, Xn suppressed glycolysis in human colorectal cancer cells by downregulating the epidermal growth factor receptor (EGFR)-AKT pathway and the expression of HK2. Xn reduced glucose consumption and lactate production and elevated the culture medium pH, indicating attenuated extracellular acidification that may ameliorate the acidic TME and limit metastasis (Liu Y. et al., 2019).

### 5.4 Quinones

#### 5.4.1 Shikonin (SK)

Shikonin (SK), a natural naphthoquinone pigment isolated from *Lithospermum* roots, primarily comprises lithospermic acid and its derivatives. This compound possesses anticancer, anti-inflammatory, antibacterial, and analgesic properties (Guo et al., 2019; Sun et al., 2022). SK functions as an inhibitor of PKM2, an enzyme involved in the regulation of both glycolysis and OXPHOS. Research on the combination of SK and olaparib (Ola) showed that at concentrations of 4, 6, or 8 μM, SK suppresses PKM2 and thus enhances the antitumor effect of Ola in OC cells. Xenograft animal models confirmed that the combined administration of SK and Ola effectively inhibits tumor development. Furthermore, examinations of tissue samples indicated that treatment with SK reduced the expression of breast cancer gene 1 (BRCA1) and PKM2. These findings indicate that SK and Ola synergistically target OC through PKM2 inhibition (Zhou S. et al., 2022). Additionally, SK decreases the levels of glucose intake, lactate generation, ECAR, and OC through the downregulation of PKM2 (Chao et al., 2017).

#### 5.4.2 KA-4s

KA-4s, a derivative of anthraquinone, is a product of artificial chemical modification rather than a naturally occurring compound. Its parent structure belongs to the anthraquinone family, which occurs ubiquitously in various plants and traditional Chinese medicinal products, such as *Rubia cordifolia* and *Rheum palmatum* (e.g., alizarin and emodin). Derivatives, such as KA-4s, possess potential application value in antitumor therapy (Zhai et al., 2023; Zhao et al., 2024a; Mishra et al., 2025). Specifically, the treatment of SKOV3 and SKOV3/DDP cells with 1 μM–7 μM kA-4s induced a concentration-dependent downregulation of GLUT1 and GLUT4 expression in both cell lines. The OCR progressively decreased in response to increasing concentrations of KA-4s, while reductions were also observed in maximal respiration, basal respiration, ATP production, and reserve respiratory capacity. Furthermore, cells treated with KA-4s exhibited lower glycolytic proton efflux rates, OCR, and ECAR values than control cells. In an orthotopic OC xenograft model, KA-4s treatment suppressed the expression of GLUT1 and GLUT4. These findings indicate that KA-4s inhibits the activity and proliferation of SKOV3/DDP and SKOV3 cells by suppressing glycolytic activity (Zhao et al., 2024b).

#### 5.4.3 Emd-D

Emd-D is a rhein derivative designed to exhibit enhanced pharmacological properties and bioavailability compared with that of the parent compound. Additionally, it possesses antiviral, anti-inflammatory, and anticancer properties (Narender et al., 2013; Dong et al., 2016; Liu et al., 2021). Mechanistic investigations showed that Emd-D exerts its inhibitory effects through direct binding with the glycolysis-associated enzyme 6-phosphofructo-2-kinase/fructose-2,6-bisphosphatase 4 (PFKFB4). Treatment with varying doses of Emd-D (10, 30, and 100 μM) led to a dose-dependent reduction in intracellular lactate and pyruvate levels, along with a decrease in GLUT1 and HK2 expression in OVHM and SKOV3 cells. Furthermore, ECAR analysis demonstrated that Emd-D treatment impaired both the basal and maximal glycolytic capacities in both cell lines in a concentration-dependent manner. PFKFB4 overexpression experiments additionally confirmed that Emd-D-dependent apoptosis relies on PFKFB4-mediated glycolysis and the steroid receptor coactivator-3 (SRC-3)/mTOR complex 1 (mTORC1) pathway. *In vivo*, Emd-D treatment decreased tthe umor volume, inhibited glycolysis, and enhanced the expression of the apoptotic proteins BCL2-associated X protein (BAX) and B-cell lymphoma 2 (BCL-2). Collectively, these data indicate that Emd-D exerts antitumor effects through the suppression of PFKFB4-fueled glycolysis and the simultaneous promotion of apoptosis (Zhao et al., 2025).

### 5.5 Terpenoids

#### 5.5.1 Triptolide (TP)

Triptolide (TP), a terpenoid compound isolated from the celastraceous plant *Tripterygium wilfordii*, possesses antioxidant, anti-rheumatic, anti-dementia, and anticancer properties (Gao et al., 2021; Song et al., 2023). HK2 is an essential protein in the glycolytic pathway. In A2780/CP70 cells, TP significantly reduced HK2 protein levels following cisplatin treatment, but it exerted only minimal influence on HK2 expression in cisplatin-sensitive A2780 cells. These results indicate that TP antagonizes cisplatin resistance in human OC cells through the downregulation of HK2 expression, with a markedly more pronounced effect observed in A2780/CP70 cells than in A2780 cells (Wang et al., 2018). Ding et al. demonstrated that TP effectively inhibits the growth of patient-derived organoids and suppresses OC progression by attenuating exosomal integrin alpha-5 (ITGA5)-induced migration of human peritoneal mesothelial cells and mitigating lactate-driven metabolic reprogramming (Ding et al., 2025). Conversely, a different study showed that TP treatment promotes the concentration-dependent accumulation of HIF-1α protein while simultaneously increasing HIF-1α mRNA levels (Zhou et al., 2010).

#### 5.5.2 Celastrol

Celastrol, a natural pentacyclic triterpenoid isolated from the root bark of *Celastrus oregonensis* (family Celastraceae), displays anti-inflammatory, antitumor, lipid-regulating, and anti-rheumatoid activities (Venkatesha et al., 2016; Xu et al., 2021; Wang C. et al., 2023). The treatment of A2780 cells with 100, 500, and 1,000 nM celastrol led to the dose-dependent suppression of HIF-1α and Myc protein expression. Furthermore, RT-PCR and Western blot analyses demonstrated that celastrol downregulates the mRNA expression of Myc, cell-division cycle 37 (CDC37), and fibronectin 1 (FN1). Both HIF-1α and Myc play essential roles in glycolysis (Wang et al., 2022).

#### 5.5.3 Fucoxanthin

Fucoxanthin is a natural pigment belonging to the xanthophyll class of carotenoids. It serves as a colorant in Phaeophyceae, Bacillariophyta, and Chrysophyta, and it is common in numerous algae, marine phytoplankton, aquatic shellfish, flora, and fauna (Pang et al., 2024). This compound demonstrates pharmacological properties, including antitumor, anti-inflammatory, antioxidant, weight loss, and neuroprotective effects (Liu X. et al., 2020; Méresse et al., 2020; Xiao et al., 2020; Pang et al., 2024). In experiments where A2780 and OVCAR3 cells were exposed to fucoxanthin at concentrations of 50, 75, and 100 μM, increased concentrations of fucoxanthin led to decreased utilization of [^18^F]fluoro-2-deoxy-D-glucose (FDG). Moreover, the ATP levels fluctuated in response to the fucoxanthin concentration, while the expression of LDHA, HK2, and PDK1 was substantially downregulated. Meanwhile, fucoxanthin suppressed the phosphorylation of STAT3 and c-Myc protein expression. These results collectively indicate that fucoxanthin inhibits glucose metabolism in OC cells by modulating the STAT3/c-Myc signaling pathway (Zhang et al., 2023).

#### 5.5.4 Isoalantolacton (ISO)

Isoalantolacton (ISO), a sesquiterpene lactone found in the roots and leaves of plants such as *Artemisia argyi* and *Artemisia sinensis* (Asteraceae family), possesses apoptosis-inducing properties and exhibits potent cytotoxic activity against certain cancer cell lines (Rasul et al., 2013; Yang C. et al., 2024). ISO can overcome cisplatin resistance and serves as a potential inhibitor of glycolysis. The restraining effect of ISO on the glycolytic pathway in cisplatin-resistant OC cells primarily involves the upregulation of LDHA, PFKL, citrate synthase (CS), pyruvate dehydrogenase E1 subunit alpha 1 (PDHA1), HK2, and GLUT1, alongside the downregulation of phosphoglycerate dehydrogenase (PHGDH). This activity reduces glucose consumption and the production of extracellular lactate in A2780^cisR^ and SNU-8^cisR^ OC cells. The susceptibility of OC cells to chemotherapy is increased *via* ISO. Combination therapy of ISO and cisplatin inhibits the MAPK/AKT pathway in cisplatin-resistant cells more effectively than either agent alone. Studies in A2780^cisR^ and SNU-8^cisR^ xenograft nude mice showed that ISO suppresses tumor growth. Based on the above results, ISO may be a new class of inhibitors of glycolysis that can overcome cisplatin resistance (Chun, 2023).

#### 5.5.5 Paclitaxel (PTX)

Paclitaxel (PTX) is a good general natural product in the diterpene alkaloid class that has shown anti-cancer activity. Originally isolated and purified from the bark of the gymnosperm *Taxus chinensis*, PTX is widely used in the clinical treatment of breast cancer, OC, lung cancer, and certain head and neck cancers (Zhu and Chen, 2019; Yu et al., 2025). Compared with parental cells, PTX-resistant OC cells exhibit distinct metabolic profiles characterized by elevated OCRs, reduced intracellular ATP and guanosine triphosphate (GTP) influx rates, and increased NADH/NAD^+^ ratios. Studies indicate that HK2 is upregulated in PTX-resistant OC cells. Treatment with HK2-degrading agents was shown to reinstate PTX sensitivity and improve survival rates and OCR during PTX treatment (Lin Y. et al., 2024). Furthermore, the knockout of PFKFB2 can inhibit OC clonal growth and enhance PTX sensitivity, while siPFKFB2 transfection has been shown to increase glycolytic rates (Yang et al., 2019). The expression of KH RNA-binding domain-containing, signal transduction-associated 3 (KHDRBS3) is upregulated in PTX-resistant cells. In A2780-R and SKOV3-R cells, KHDRBS3 knockout inhibited cell proliferation, glycolysis, and colony formation *in vitro*, while also reducing the PTX IC_50_. The knockout of KHDRBS3 also enhanced PTX sensitivity in an *in vivo* xenograft model (Wu et al., 2022). Compared with the parental SKOV3 cell line, SKOV3-TR30 cells exhibit increased glycolytic flux and impaired mitochondrial function. The expression of the glycolytic enzyme phosphoglycerate mutase 1 (PGAM1) is elevated in this taxane-resistant line. In SKOV3-TR30 cells, the downregulation of PGAM1 diminished PTX resistance, while the upregulation of PGAM1 in SKOV3 exerted the opposite effect. Analysis of glycolytic flux demonstrated that the PGAM1-mediated production of pyruvate and extracellular lactate influences the resistance of OC cells to PTX (Feng et al., 2022).

#### 5.5.6 Cryptotanshinone (CT)

Cryptotanshinone (CT) is a liposoluble, bioactive diterpenoid quinone isolated from *Salvia miltiorrhiza*. This molecule exhibits anticancer, antioxidant, anti-inflammatory, and antibacterial activities (Ashrafizadeh et al., 2021; Li J. et al., 2021). The treatment of A2780, CAOV3, and IOSE80 cells with 0 μM–20 μM CT reduced glucose utilization and lactate production in a dose- and time-dependent manner, while simultaneously inhibiting the activity of HK2 and LDHA. Molecular docking analyses demonstrated that CT directly binds to PKM2, LDHA, HK2, GLUT1, and complex I. Furthermore, immunoblotting results indicated that CT treatment diminished the production of aberrantly highly expressed glycolytic proteins, namely, GLUT1, HK2, PKM2, and LDHA. Xenotransplantation models further confirmed the results of the *in vitro* experiments. Combined, the *in vitro* and *in vivo* findings indicate that CT reduces ATP levels, inhibits HK2 and LDHA activity, and suppresses the expression of the glycolysis-related proteins LDHA, GLUT1, and HK2 within tumor tissues. CT was also reported to block the Warburg effect and OXPHOS in OC cells, thereby suppressing cell proliferation and inducing apoptosis (Wang T. et al., 2024). Another study revealed that CT inhibits cellular glucose consumption and lactate generation through the suppression of the STAT3/sirtuin 3 (SIRT3)/HIF-1α signaling pathway, leading to the downregulation of GLUT1, LDHA, and HK expression (Yang Y. et al., 2018). Additionally, the CT-induced reduction in extracellular lactate observed in both studies may ameliorate the TME.

#### 5.5.7 Tanshinone I (Tan I)

Tanshinone I (Tan I), a natural diterpene quinone isolated from *S. miltiorrhiza*, is structurally similar to tanshinone IIA. This compound exhibits a wide spectrum of biological activities, including anti-inflammatory, antioxidant, anticancer, and neuroprotective effects (Yang L. et al., 2021; Huang et al., 2022; Roth et al., 2023). Treatment of A2780 and ID-8 cells with Tan I (0.5, 5, and 50 μg/mL) suppressed the expression of genes encoding the glycolysis-related proteins HK2, PFK, enolase 2 (ENO2), and LDHA in a dose- and time-dependent manner, leading to reduced lactate formation and levels of H3K18la and, consequently, the suppression of the expression of tumor-associated threonine tyrosine kinase, platelet-derived growth factor receptor β (PDGFRβ), YTH domain family 2 (YTHDF2), and rubicon-like autophagy enhancer (RUBCNL). In xenograft tumors, Tan I treatment increased intra-tumoral CD4^+^ and CD8^+^ T cells, decreased Tregs, and reduced M2-like tumor-associated macrophages, which indicated an improved tumor immune microenvironment (Jin Z. et al., 2025).

### 5.6 Alkaloids

#### 5.6.1 NK007

NK007, a (±)-tyramine malate derivative isolated from the Asclepiadaceae plants, possesses antitumor and anti-inflammatory properties (Wen et al., 2012a; Wen et al., 2012b; Chen et al., 2015; Wang S. et al., 2021). Treating A2780, A780 (PTX), 2774-C10, OVCAR3, and H08910 cells with NK007 (0 μM–200 μM) suppressed HK2 protein expression and reduced glucose uptake, OCR, and ECAR. According to the above data, NK007 suppresses PTX resistance by inhibiting glycolysis through the reduction of HK2 (Li et al., 2019).

#### 5.6.2 Berberine (BBR)

Berberine (BBR) is a quaternary ammonium alkaloid that can be obtained from the rhizome of the Chinese medicinal herb *Coptis chinensis*. This compound exhibits a broad range of pharmacological activities, including anti-inflammatory, antibacterial, anticancer, cardioprotective, hypoglycemic, lipid-regulating, and immunosuppressive effects (Feng et al., 2019; Zhou W. et al., 2025). Furthermore, BBR serves as an effective insulin sensitizer (Rondanelli et al., 2021). To determine its effects on glycolysis in OC cells, HEY cells were exposed to 10 and 15 μM BBR, while SKOV3 cells were treated with 20 and 40 μM BBR. The results showed that BBR modulates OC cell glycolysis in a concentration-dependent manner. Specifically, both the OCR and the ECAR were significantly reduced across the various BBR concentrations. PCR and Western blot analysis demonstrated that BBR concentration-dependently suppressed the expression of the glycolysis and autophagy-related molecules GLUT4, LDHB, and HK2 and the microtubule-associated protein 1 light chain 3 (LC3)A/B in OC cells. Furthermore, the knockout of LINC01123 and P65 abolished these effects, indicating that BBR modulated glycolysis and autophagy in OC cells by targeting the LINC01123/P65/MAPK10 signaling pathway. Similarly, *in vivo* experiments showed that BBR modulated the levels of autophagy and glycolysis in OC cells by activating the LINC01123/P65/MAPK10 pathway (Yan et al., 2024). Additionally, BBR exposure increased TET3-mediated demethylation, promoted the miR-145-mediated inhibition of HK2, and decreased lactate production and glucose usage, which ultimately abrogated glycolysis in OC cells (Li H. et al., 2021). In a peritoneal metastasis model, BBR treatment increased intra-tumoral CD4^+^ and CD8^+^ T-cell infiltration, reduced PD-L1 and NF-κB expression, alleviated adipose tissue senescence with decreased IL-1β and CXCL2 levels, inhibited lipid metabolism, and promoted the proliferation of tumor-infiltrating immune cells, thereby ameliorating the immunosuppressive TME and suppressing OC metastasis (Zhang Z. et al., 2025).

### 5.7 Glycosides

#### 5.7.1 Sulforaphane (SFN)

Sulforaphane (SFN) is derived from a glucosinolate precursor, glucoraphanin, through conversion by myrosinase, an enzyme primarily found in cruciferous plants. SFN exhibits significant anticancer, antioxidant, anti-inflammatory, and antiviral activities (Janczewski, 2022; Otoo and Allen, 2023). In a study involving the A2780 OC cell line and two resistant variants (A2780/ADR and A2780/CP), treatment with SFN (2.5, 5, and 10 μM) reduced HIF-1α protein levels. This treatment simultaneously decreased both the transcriptional and protein expression of the HIF-1 targets carbonic anhydrase IX (CA IX) and GLUT 1. By suppressing the HIF-1α/CA IX axis, SFN disrupted pH regulation under hypoxia, as evidenced by decreased intracellular pH and increased extracellular pH, indicating amelioration of the acidic TME. Disruption of OC cells’ migration inhibited chemoresistance in these cells to a certain extent (Pastorek et al., 2015).

#### 5.7.2 Salidroside (SAL)

Salidroside (SAL) is derived from the roots and rhizomes of *Rhodiola rosea*. Its wide-ranging biological activities include antitumor, immunomodulatory, and antioxidant effects (Chiang et al., 2015; Pu et al., 2020; Zhang Q. et al., 2021; Zhang X. et al., 2021). In OC, SAL targets the STAT3/c-Myc axis, a primary signaling pathway in the regulation of glycolytic flux (Sun et al., 2021; Gao et al., 2023). Treatment of SKOV3, CAOV3, and IOSE80 cells with SAL (50 μM–800 μM) for 24 h inhibited the STAT3/c-Myc pathway and downregulated the expression of glycolysis-related proteins both *in vitro* and *in vivo*. The above phenomena are not concentration-dependent. SAL inhibited the expression of epithelial–mesenchymal transition factors and significantly reduced the expression of the essential glycolytic enzyme system LDHA, GLUT1, and HK2. These molecular changes reduced glucose consumption, decreased the production of extracellular lactate, and lowered ATP generation; thus, ECAR was reduced in CAOV3 and SKOV3 cells. Finally, SAL inhibited the STAT3/c-Myc-driven glycolytic pathway to suppress tumor cells (Yu and Feng, 2024).

#### 5.7.3 Ginsenoside 20(S)-Rg3 (20(S)-rg3)

20(S)-Rg3 is a highly active ginsenoside and one of the primary bioactive compounds derived from ginseng. It exhibits various pharmacological properties, including anti-ischemic, hypoglycemic, anti-allergic, and anti-inflammatory activities, along with prominent antitumor effects (Nakhjavani et al., 2020; Xiaodan and Ying, 2022; Guan and Qi, 2023). In OC models, treatment with 20(S)-Rg3 (160 μg/mL for SKOV3 cells and 80 μg/mL for 3AO cells) inhibited the expression of the key glycolytic enzymes GLUT1, HK2, LDH, PKM2, PDK, and PFK. Furthermore, 20(S)-Rg3 was reported to modulate HK2 expression by downregulating the phosphorylation of STAT3 at Tyr705. In reverse validation experiments, STAT3 overexpression was found to upregulate glucose utilization and lactate production, which abrogated the inhibitory effects of 20(S)-Rg3. *In vivo* experiments further demonstrated that 20(S)-Rg3 suppressed HK2 expression in xenograft models, confirming that it inhibited glycolysis by targeting the STAT3/HK2 pathway (Li et al., 2015). Furthermore, Lu et al. (2019), Lu et al. (2020) reported that 20(S)-Rg3 suppressed glycolysis—characterized by reduced glucose utilization, lactate production, and HK2 expression—by upregulating miR-603 expression. Another study established that 20(S)-Rg3 upregulated the expression of miR-519a-5p, which subsequently targeted and inhibited HIF-α, thereby silencing the Warburg effect in OC cells. Similarly, Zheng et al. (2018) indicated that 20(S)-Rg3 enhanced the inhibitory effects of miR-324-5p on glucose utilization and PKM2 expression by blocking the HIF-1α-mediated suppression of this microRNA.

#### 5.7.4 Bufalin

Bufalin is a cardiotonic steroid extracted from the venom of Bufonidae amphibians. It exhibits significant antitumor activity, inhibits vascular endothelial cell proliferation, and suppresses angiogenesis (El-Seedi et al., 2022; Soumoy et al., 2022; Asrorov et al., 2023). In OC-related studies, the treatment of A2780 and HEY cells with bufalin (5 and 10 nM) for 24 h significantly reduced glucose uptake and extracellular lactate production compared to that in control groups. Furthermore, bufalin downregulated the expression of the glycolysis-related proteins GLUT4, LDHB, and HK2. *In vivo* results from xenografted nude mice were consistent with these *in vitro* findings, confirming that bufalin inhibited cell growth and proliferation by suppressing the integrin beta 2 subunit/focal adhesion kinase (ITGB2/FAK) signaling pathway (Li X. et al., 2018).

### 5.8 Phenylpropanoids

#### 5.8.1 Dicumarol (DIC)

Dicumarol (DIC) is a natural anticoagulant typically sourced from plants such as vanilla, cardamom, and nutmeg. While traditionally known for its ability to antagonize vitamin K, recent studies confirmed that DIC and its derivatives possess anti-inflammatory, antibacterial, and antitumor properties (Garg et al., 2020; Sun X. et al., 2020; Ge et al., 2024). Exposing SKOV3 and A2780 cells to DIC (100, 200, and 300 μM) dose-dependently inhibited PDK1 activity and led to a significant reduction in glucose uptake and extracellular lactate production. The inhibition of PDK1 led to a significant increase in the OCR and a concomitant reduction in the ECAR. Furthermore, DIC treatment increased the levels of ROS and reduced the mitochondrial membrane potential (MMP). *In vivo* experiments recapitulated this phenotype, as evidenced by the significant reduction in tumor growth observed in xenografted mice after DIC treatment. Further analysis showed that the levels of phosphorylated PDHA1 were decreased in the mice, indicating that DIC suppressed PDK1 kinase activity *in vivo*. These metabolic changes shifted glucose metabolism toward OXPHOS and led to increased ROS levels, decreased MMP, and the stimulation of apoptosis (Zhang et al., 2017).

#### 5.8.2 Agrimonolide (AM)

Agrimonolide (AM) is a bioactive isocoumarin primarily extracted from the roots and rhizomes of the rose plant *Agrimonia pilosa*. Its pharmacological properties include the reduction of smooth muscle spasms, hepatoprotection, and anti-inflammatory, hypoglycemic, and antitumor activities (Chen L. et al., 2016; Park and Kang, 2020; Liu et al., 2022; Huang et al., 2023; Han et al., 2025). In OC models, the treatment of SKOV3 and A2780 cells with AM (10, 20, and 40 μM) dose-dependently inhibited glucose uptake and extracellular lactate production. This metabolic shift correlated with significant reductions in the protein expression levels of HIF-1α, HK2, and LDHA. Furthermore, ECAR analysis confirmed that glycolytic activity declined following AM treatment. These inhibitory effects were reversed following HIF-1α overexpression, indicating that AM suppressed glycolysis in OC cells *via* the downregulation of the HIF-1α axis (Yang Y. et al., 2024).

### 5.9 Steroids

#### 5.9.1 *Paris* saponins VII (PS VII)

*Paris* saponins VII (PS VII) is a steroidal saponin isolated from the rhizomes of *Paris* species, such as *Paris polyphylla* and *Paris tetragona*. It exhibits a wide range of pharmacological activities, including antibacterial, antiviral, anti-inflammatory, and antitumor effects (Thapa et al., 2022; Xiang et al., 2022; Zhang Y. et al., 2024). In OC models, treatment with PS VII (2 and 4 μM) effectively suppressed glycolytic activity and lowered both the OCR and ECAR in PARP inhibitor-resistant (PARPi-R) SKOV3 and HEY cells. When the expression of the retinoic acid receptor-related orphan receptor alpha (RORα) was downregulated, the inhibitory effects of PS VII on glycolysis were reversed. *In vivo* experiments further demonstrated that PS VII, administered either alone or in combination with PARP inhibitors, modulated key molecules associated with the RORα/extracellular matrix protein 1 (ECM1)/vascular endothelial growth factor receptor-2 (VEGFR2) signaling axis in nude mice, consistent with the above findings (Wang M. et al., 2024). Wu et al. demonstrated that PS VII increased the percentage of apoptotic cells in a dose-dependent manner. This treatment inhibited glucose utilization and lactate, ATP, and nicotinamide adenine dinucleotide phosphate hydrogen (NADPH) generation in both SKOV3IP1 and HEY cells. Based on the above results, OCR and ECAR measurements show that PS VII-treated cells have a reduced glycolytic phenotype and significantly lower glycolytic capacity. Concurrently, PS VII elevated retinoic acid receptor-related orphan receptor alpha (RORC) expression in a dose-dependent manner. Notably, PS VII decreased the increase in glucose utilization, extracellular lactate production, and ATP/NADPH generation caused by RORC downregulation. In addition, the overexpression of activated CDC42-associated kinase 1 (ACK1) also reversed these metabolic effects; thus, PS VII inhibits glycolysis and promotes apoptosis through the RORC/ACK1 signaling pathway (Wu Z. et al., 2023).

#### 5.9.2 Melatonin (Mel)

Melatonin (Mel) is an indoleamine hormone found in the pineal gland, various plants (such as cherries), and bacteria. It is involved in diverse physiological processes, including the regulation of sleep–wake cycles, antioxidant defense, immunomodulation, and tumor suppression (Minich et al., 2022; Ahmad et al., 2023; Joseph et al., 2024; Reiter et al., 2024). In OC models, Chuffa et al. (2016) observed that long-term Mel treatment significantly reduced HIF-1 signaling and suppressed broader cellular metabolic pathways, including those associated with pyruvate metabolism, glycolysis, gluconeogenesis, and the respiratory electron transport chain. Specifically, treating SKOV3 and CAISMV-24 cells with Mel (2.0 μM–7.0 μM) for 24 h lowered the expression and activity levels of several key metabolic enzymes, including LDH, HIF-1α, CS, and PDH. Furthermore, Mel reduced the levels of glyceraldehyde-3-phosphate dehydrogenase (GAPDH) and glucose-6-phosphate dehydrogenase (G6PDH) in both cell types. Mel treatment significantly reduced the activity of enzymes associated with the Warburg effect and glutamine catabolism, including glutamine synthetase (GS), LDH, CS, G6PDH, and PFK-1. Conversely, Mel increased the activity of PDH, reflecting a metabolic shift from fermentation to mitochondrial OXPHOS. In addition, Mel decreased extracellular lactate production and glutamine levels in both cell lines, which may limit lactate efflux and ameliorate the TME acidification, thereby contributing to the suppression of OC progression. These findings demonstrated that Mel reversed Warburg metabolism and reduced glutamine catabolism, thereby attenuating the influence of multiple oncogenic molecules involved in OC progression and invasiveness (Silveira et al., 2024). Furthermore, in models of environmental toxicity, Pal et al. demonstrated that Mel stimulated the expression of key redox and survival markers by upregulating the silent information regulator 1 (SIRT1)/nuclear factor erythroid 2-related factor 2 (Nrf2)/nuclear factor kappa-B (NF-κB) and insulin resistance (IR)/PI3K/p-AKT/GLUT4 signaling pathways. In aged hamsters exposed to bisphenol S (BPS), Mel enhanced the ovarian antioxidant capacity and reduced the inflammatory burden, thereby ameliorating the resultant inflammation and metabolic alterations (Pal et al., 2023).

### 5.10 Macrolides

#### 5.10.1 Ivermectin (IVM)

Ivermectin (IVM) is a semi-synthetic macrolide polycyclic antibiotic derived from avermectin, a compound isolated from the fermentation products of *Streptomyces avermitilis*. While traditionally utilized as a broad-spectrum antiparasitic, recent studies confirmed that IVM shows anti-inflammatory, antiviral, and antitumor activities (Draganov et al., 2015; Mathachan et al., 2021; Patel et al., 2025). Quantitative proteomics of clinical OC tissues shows that endometrioid OC tissues are produced at an increased rate *via* aerobic glycolysis. TOV-21G cell models and the corresponding tumor-bearing mouse xenograft models showed that IVM suppressed glycolysis. In addition, the expression of monocarboxylate transporters MCT1 and MCT4 was increased in OC tissues and suppressed by IVM treatment. Given that MCT1 and MCT4 mediate lactate efflux and contribute to extracellular acidification, their downregulation may attenuate TME acidosis and relieve the associated immunosuppression (Li N. et al., 2020). Compared to control treatments, IVM significantly reduced the protein levels of several key metabolic enzymes, including PFKP, PKM, dihydrolipoamide dehydrogenase (DLD), and dihydrolipoamide S-acetyltransferase, in OC tissues (Li et al., 2024).

### 5.11 Proteins

#### 5.11.1 MAP30

MAP30 is a 30 kDa single-chain type 1 ribosome-inactivating protein derived from bitter melon (*Momordica charantia*). It possesses diverse biological properties, including antiviral and antibacterial effects, in addition to significant antitumor effects (Dandawate et al., 2016; Chan et al., 2020; Wang Z. et al., 2021). MAP30, a primary bioactive component of bitter melon extract (BME), was found to act as a potent AMPK activator, suppressing the growth, migration, and invasion of OC cells (Yung et al., 2016a; Yung et al., 2016b). Studies have shown that MAP30 treatment (5 μg/mL–40 μg/mL) dose-dependently inhibited the migration, invasion, and proliferation of A2780cp, OVCA433, and ES2 cells. Notably, these inhibitory effects were not observed in normal immortalized ovarian epithelial cells (HOSE11-12). Furthermore, when administered in combination with cisplatin, MAP30 exhibited significant cytotoxic synergism in cisplatin-resistant OC cells. By activating the AMPK signaling axis, MAP30 suppressed elevated lactate production and inhibited the enzymatic activities of HK2 and LDH. It also reduced glucose uptake in a dose-dependent manner and downregulated the expression of the glucose transporters GLUT1 and GLUT3 over time. This glycolytic attenuation was accompanied by reduced total fatty acid levels in malignant ascites and the reversal of omental conditioned medium-induced lipid droplet accumulation. These metabolic changes led to a marked decrease in ATP production, confirming that MAP30 blocked glycolytic flux in OC cells through the activation of AMPK. Collectively, these findings indicate that MAP30 remodels the nutrient landscape of the peritoneal TME, thereby contributing to the suppression of tumor dissemination *in vivo* (Chan et al., 2020).

In short, 33 natural compounds from different chemical categories have been shown to inhibit OC glycolysis through multiple mechanisms, such as limiting the glucose uptake, inhibiting the PI3K/AKT/mTOR-HIF-1α pathway, reducing glycolytic enzyme expression (HK2, PFK, PKM2, and LDHA), and decreasing lactate generation. Many of them also alter the TME by reducing lactate-driven immunosuppression. Tables 1 and 2 summarize their models, mechanisms, and TME-related effects.

### 5.12 Comparison and critical discussion

Although all 33 natural compounds listed above inhibit glycolysis in OC, they differ in terms of potency, selectivity, drug-likeness, and translational readiness. All these studies have shown some characteristics of the chemical groups and what needs to be addressed.

#### 5.12.1 Efficacy and potency

Different half-maximal inhibitory concentrations (IC_50_) or effective concentrations are required for direct glycolytic inhibition. For example, TP is a potent anti-glycolytic agent at the nanomolar level (IC_50_ = 40 nM in SKOV-3 cells) (Wu et al., 2020), and celastrol inhibits the proliferation of A2780 cells in a dose-dependent manner at 0.1 μM–1 μM (Wang et al., 2022); on the other hand, polyphenols such as RV and Pae require much higher concentrations (RV 200 μM and Pae 500 μM) to achieve a similar suppression of glucose uptake or lactate production (Liu et al., 2018; Yang et al., 2025). The difference of almost 1,000 times indicates that terpenoids and some quinones (e.g., SK) may have a relatively wide therapeutic window, but their higher potency often comes at the cost of increased off-target toxicity (see below). Flavonoids (e.g., QR and phloretin) and glycosides (e.g., SAL) show micromolar activity in the intermediate range. Not all of the potency shown *in vitro* will be manifested in the same manner after *in vivo* administration due to changes in pharmacokinetics.

#### 5.12.2 Selectivity for cancer vs. normal cells

Most of the reviewed compounds have been tested mainly on OC cell lines and have had little comparison with non-malignant ovarian epithelial cells (e.g., IOSE80). A few studies have provided some data: CBD was not significantly toxic to normal immortalized ovarian epithelial cells (Tong et al., 2025), and MAP30 also showed no obvious cytotoxicity in normal HOSE11-12 cells (Chan et al., 2020); thus, it is relatively selective. In contrast, oleic acid actually promoted proliferation and glycolysis in OC cells (Kado et al., 2023), raising concerns about its use in OC patients. For many compounds, selectivity data remain absent, making it difficult to assess their therapeutic index.

#### 5.12.3 Bioavailability

As discussed in Section 6, poor aqueous solubility and extensive first-pass metabolism plague many natural products. Among the reviewed compounds, berberine has been documented to have an approximately 5%–10% gut absorption rate (Wang et al., 2019). Resveratrol also suffers from extremely low oral bioavailability in humans, as confirmed by a meta-analysis of 84 oral administrations (Szymkowiak et al., 2025). Cryptotanshinone and tanshinone I, although lipophilic, suffer from poor aqueous solubility and low oral bioavailability (Chen M. et al., 2016; Xing et al., 2017). Semi-synthetic derivatives such as KA-4s and Emd-D have been designed to improve these properties. Future development will likely require formulation technologies (nanoparticles and liposomes) for most of these compounds.

#### 5.12.4 Toxicity and safety

Systematic toxicity comparisons are rarely performed within a single study. Nevertheless, certain compounds have well-known safety concerns: triptolide can cause hepatotoxicity and nephrotoxicity at doses only slightly above its therapeutic range (Song et al., 2023). Celastrol has been associated with liver injury, kidney toxicity, and cardiovascular and hematopoietic system side effects (Li et al., 2022; Jiang et al., 2026). Shikonin may potentially cause nephrotoxicity and skin allergy (Sun et al., 2022). On the other hand, dietary compounds such as resveratrol have generally favorable safety profiles in humans (doses up to 1 g/day are generally well tolerated) (Brown et al., 2024). The review would be strengthened if future studies explicitly report cytotoxicity against non-cancerous cells and sub-acute toxicity data in animal models.

#### 5.12.5 Translational potential

There are some problems in the conversion of these natural glycolysis inhibitors to OC therapy. However, the prospects are still relatively limited. Chemosensitizing effects (e.g., corilagin, isoalantolactone, NK007, and berberine with cisplatin/PARP inhibitors) have been studied to develop rational combination regimens (Jia et al., 2017; Li et al., 2019; Chun, 2023; Zhang X. et al., 2025). Multi-target actions (e.g., tanshinone I reduces lactate, H3K18la, and TME immunosuppression) and favorable safety profiles of natural compounds are also drawing attention (Jin B. et al., 2025), and advanced formulations such as nanoparticles and liposomes may overcome the barriers of bioavailability. Predictive biomarkers (HK2, LDHA, and PKM2) may enable patient stratification. Although they have certain deficiencies at present, natural glycolysis inhibitors are promising and relatively unexplored directions for formulation development and biomarker-guided studies.

In short, although the 33 natural compounds as a group support the idea of targeting glycolysis in OC, detailed studies have shown significant differences in their strength, specificity, and other drug-like properties. Future research should move beyond descriptive reporting toward mechanistic comparison, systematic toxicity assessment, and rational combination strategies.

## 6 Bottlenecks in the therapeutic translation of natural compounds

Recently, there have been many studies abroad on the development of anticancer drugs based on natural products, and many issues still need to be resolved before such drugs can be applied to human treatment. The following are the main shortcomings.

### 6.1 Extraction strategies and yield optimization

Natural products are generally obtained from all kinds of plants, animals, and microbes, and they have complex chemical structures that are difficult to extract and purify. The composition and concentration of the final product will be different according to the extraction and purification methods utilized and whether a new source or a cumulative one has been utilized over time. The above factors are likely to affect batch-to-batch consistency and, thus, prevent the standardization of quality control in clinical-grade production (Wilson et al., 2020). The quality will be unstable, and thus, there is no guarantee of the safety and efficacy of the drugs for patients; harm will be inflicted on patients during clinical use, and industrial production will be interrupted. In addition, as the components and origins of drugs become more complex, the cost and time to develop them increase accordingly; thus, the rapid progress and broad application of natural anticancer drugs have been restricted. Crucially, there are no standardized reference materials available; thus, the same compound studied by different groups may vary significantly, leading to compromosed reproducibility and cross-study comparison.

### 6.2 Physicochemical characterization and bioavailability

Natural compounds are generally quite chemically complex. The main reasons for chemical changes in stored drugs that reduce or eliminate their therapeutic effect are often exposure to light and oxidation (Chen et al., 2022b; Luiza Koop et al., 2022). In addition, many of the highly effective natural products with anti-cancer properties have poor water solubility and are, thus, difficult to absorb and distribute throughout the body. Flavonoids and alkaloids have shown good *in vitro* activities, but their poor solubility in physiological fluids has not been fully resolved (Yang X. et al., 2021; Rocha et al., 2023; Bao et al., 2024; Liu D. et al., 2024; Gupta et al., 2026; Pei et al., 2026). Due to this reason, the molecules cannot cross the cell membranes of tumor cells effectively and are, thus, less likely to have a therapeutic effect.

Natural components of the body are broken down and absorbed in the digestive system; then, they are subjected to the first-pass effect in the liver. Metabolism of a drug can reduce its concentration at the site of action by degrading it or excreting it. For example, several terpenoid compounds have been widely degraded and transformed in the intestine and liver after oral administration (Yan et al., 2018; Kashyap et al., 2021; Georgiou et al., 2023; Ma et al., 2026). Therefore, there is a severe loss of activity, and it will be unable to reach the site of disease effectively. Many natural anti-cancer drugs also have a short half-life and are, therefore, rapidly cleared from the body (Shafi et al., 2025). If it is to be used for an extended period, more frequent administration will be required to ensure the good health of the patient and prevent harm. Protracting this may also cause harm to the patient through side effects. Variations in people’s metabolism also affect the speed of clearance and half-life; therefore, standardized treatment plans are difficult to develop. The structure that gives rise to a strong biological effect may not meet the requirements for good pharmacokinetics. Formulation technologies such as nanoparticles and liposomes have shown some promise in addressing the above problems, but their own long-term safety and manufacturing scalability are still issues that need to be resolved (Li N. et al., 2020; Singla et al., 2022; Elsebay et al., 2023).

### 6.3 Adverse effects and safety profile

Certain natural antitumor compounds exhibit severe toxicities, such as cardiotoxicity, hepatotoxicity, or nephrotoxicity, which restrict the clinical dosage and administration periods (Xiong and Guan, 2017). Antitumor drugs such as anthracycline antibiotics are toxic to the heart at high doses, and repeated administration can cause irreversible damage to the heart (Zhao et al., 2018; Mohamed et al., 2024). The structure of natural products is complex and may produce harmful metabolites. Some metabolites of certain alkaloids (Liu et al., 2017; Toh et al., 2025) are more cytotoxic than the parent compounds.

Another type of severe harm is long-term chronic toxicity, such as immunosuppression and endocrine disruption, and the evaluation system for it is lacking at present. The non-selective mechanism of action has reduced the therapeutic effect and damaged healthy tissues, thus lowering the quality of life for patients. Natural anticancer drugs are also often less dose-limiting in terms of toxicity and have strong individual differences in response; therefore, standardizing treatment has been difficult. Clinically, due to the demand for favorable safety-to-effectiveness ratios, many of these treatment drugs have not been broadly applied. Systematic long-term toxicity data are largely unavailable for most of the compounds mentioned, and thus, their risk–benefit assessment and regulatory approval are difficult.

### 6.4 Lack of *in vivo* validation and clinical translation

The probability of translating *in vitro* results into clinical oncology drugs is relatively low; therefore, extensive verification in animal studies and a three-stage process of clinical trials need to be carried out to determine safety and efficacy. Many promising “cures for cancer” based on natural compounds are still in the research stage. Although the natural compounds mentioned above are likely to inhibit glycolysis in OC cells, this conclusion is based on relatively weak cell-based experiments. Animal validation is carried out in only two-thirds of these studies, and clinical trials are almost non-existent. Therefore, these immature therapies need to be verified by extensive *in vivo* and clinical studies for safety and efficacy. In addition, most of the *in vivo* studies use xenograft models in immunodeficient mice, which cannot reproduce the immune interactions at the center of glycolysis-driven TME remodeling, thus potentially overestimating efficacy and underestimating immune-related toxicities. Phase I/II trials with pharmacokinetic and pharmacodynamic endpoints need to be promptly designed and carried out to determine the dose–exposure relationship and explore rational combinations with chemotherapy or immunotherapy.

### 6.5 Tumor heterogeneity

Significant heterogeneity exists among tumor cells in OC, in addition to dissimilarities in gene expression, metabolic traits, and signaling pathways (Veneziani et al., 2023; Pieraccioli et al., 2026; Sameni et al., 2026). Given the lack of a universal therapeutic target for OC, personalized medicine has yet to be realized. The above heterogeneity indicates that the sub-populations of tumor cells will have different sensitivities to the same natural compound; some will be highly sensitive and others will remain completely resistant (Xintaropoulou et al., 2015). Fluctuation will reduce the overall effect of natural compounds in OC treatment and make it difficult to establish a stable therapeutic mechanism. Given that some OC subtypes are more dependent on glycolysis than others, biomarker-driven patient stratification based on HK2, PKM2, or LDHA expression will need to be utilized to identify those that are most likely to respond to specific natural glycolysis inhibitors.

Given the above problems, new technologies for drug delivery and standardization of production need to be explored in combination with experiments. Only then can the full effect of natural glycolysis inhibitors be achieved in OC therapy.

## 7 Summary and prospects

OC is still a very aggressive type of cancer and has a relatively high death rate. The 5-year overall survival rates of patients with advanced OC are 10%–40% (Caruso et al., 2025). OC cells are typical examples of the “Warburg effect” and, therefore, show an increase in glucose consumption and lactate production. These cells have increased expression of many glycolytic enzymes (e.g., HK2, PKM2, PFK-1, and LDHA) and traditional oncogenic signaling molecules (e.g., p53, c-Myc, HIF-1α, and PI3K/AKT/mTOR) compared to that in normal ovarian cells; thus, enhanced glycolysis is associated with the development of OC.

**FIGURE 5:**
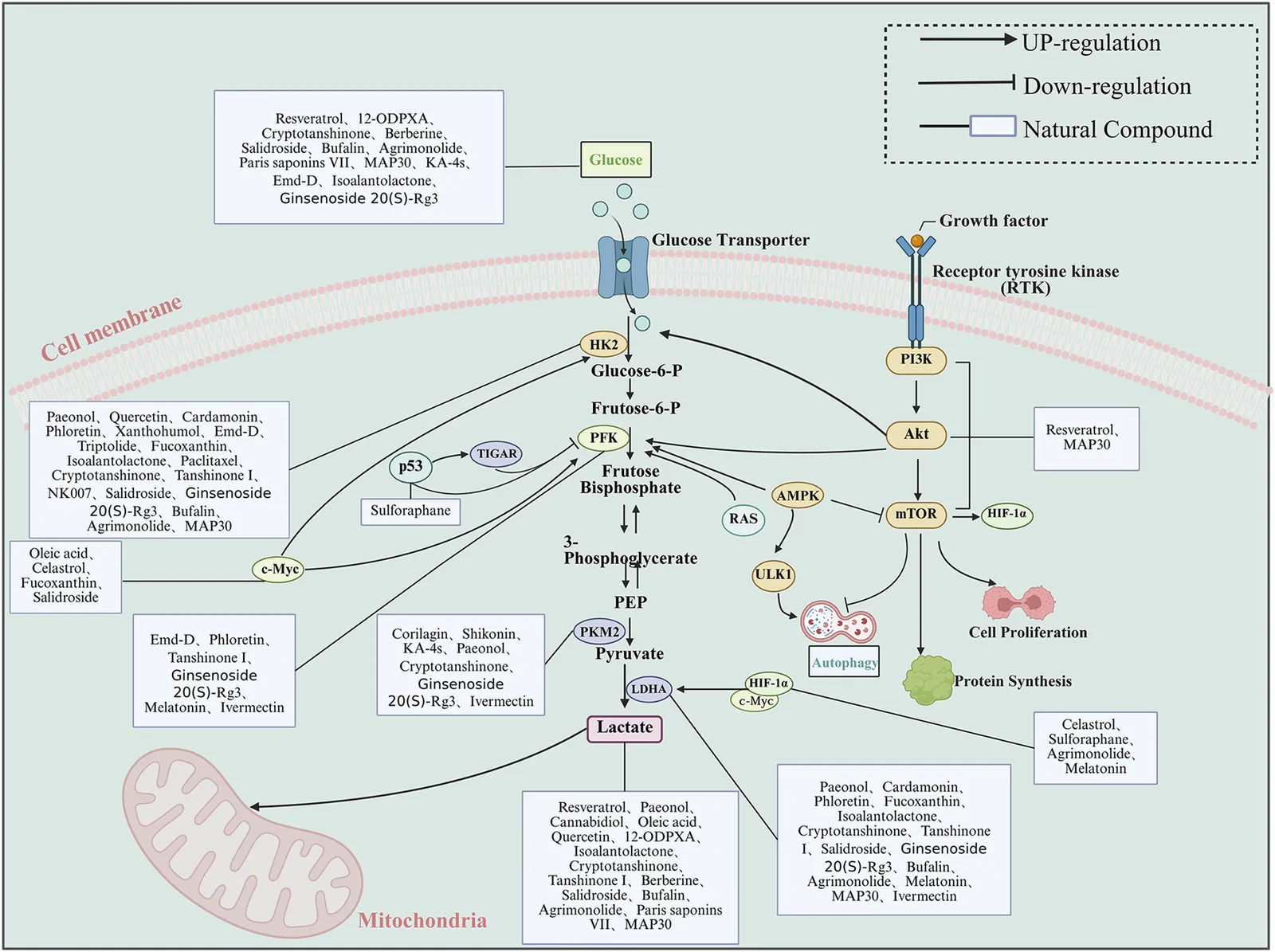
Natural compounds targeting glycolysis in ovarian cancer. All natural compounds exert anti-cancer effects *via* multi-stage, multi-target interference across glucose uptake, glycolytic enzymatic cascades, oncogenic transcription factors, proliferative kinase signaling, lactate production, cell proliferation, and protective autophagy. Most natural compounds simultaneously target multiple modules to reverse tumor Warburg effect and suppress malignant growth. Glucose uptake (cell membrane glucose transporter): resveratrol, 12-ODPXA, cryptotanshinone, berberine, salidroside, bufalin, agrimonolide, *Paris* saponins VII, MAP30, KA-4s, Emd-D, isoalantolactone, and ginsenoside 20(S)-Rg3; early glycolysis (HK2): paeonol, quercetin, cardamonin, phloretin, xanthohumol, Emd-D, triptolide, fucoxanthin, isoalantolactone, paclitaxel, cryptotanshinone, tanshinone I, NK007, salidroside, ginsenoside 20(S)-Rg3, bufalin, agrimonolide, and MAP30; tumor-suppressor p53-TIGAR axis: sulforaphane; oncogenic transcription factor c-Myc: oleic acid, celastrol, fucoxanthin, and salidroside; mid-glycolysis (PKM and pyruvate generation): corilagin, shikonin, KA-4s, paeonol, cryptotanshinone, ginsenoside 20(S)-Rg3, and ivermectin; early glycolysis (PFK): Emd-D, phloretin, tanshinone I, ginsenoside 20(S)-Rg3, melatonin, and ivermectin; late glycolysis (LDH): paeonol, cardamonin, phloretin, fucoxanthin, isoalantolactone, cryptotanshinone, tanshinone I, salidroside, ginsenoside 20(S)-Rg3, bufalin, agrimonolide, melatonin, MAP30, and ivermectin; late glycolysis (lactate accumulation): resveratrol, paeonol, cannabidiol, oleic acid, quercetin, 12-ODPXA, isoalantolactone, cryptotanshinone, tanshinone I, berberine, salidroside, bufalin, agrimonolide, *Paris* saponins VII, and MAP30; core proliferative kinase cascade (RTK-PI3K-Akt-mTOR): resveratrol and MAP30; HIF-1α-mediated glycolysis: celastrol, sulforaphane, agrimonolide, and melatonin. Created in https://BioRender.com. Abbreviations: Akt, protein kinase B; AMPK, adenosine monophosphate-activated protein kinase; c-Myc, myelocytomatosis viral oncogene homolog; Fructose-6-p, fructose-6-phosphate; Glucose-6-P, glucose-6-phosphate; HIF-1α, hypoxia-inducible factor 1 alpha; HK2, hexokinase 2; LDHA, lactate dehydrogenase A; mTOR, mammalian target of rapamycin; p53, protein 53; PEP, phosphoenolpyruvate; PFK, phosphofructokinase; PI3K, phosphatidylinositol 3-kinase; PKM2, pyruvate kinase isozyme type M2; RAS, rat sarcoma virus; TIGAR, TP53-induced glycolysis and apoptosis regulator; ULK1, unc-51-like autophagy activating kinase 1.

Among the screened natural compounds, paeonol, quercetin, cardamonin, phloretin, xanthohumol, triptolide, cryptotanshinone, and agrimonolide directly inhibit the principal glycolytic enzymes of OC cells by independent means. Other agents are resveratrol, 12-ODPXA, berberine, salidroside, bufalin, and *Paris* saponin VII; they inhibit glycolysis by reducing glucose uptake and inhibiting the production of lactate and pyruvate. Cannabidiol and BME also inhibit glycolysis indirectly by regulating PI3K/AKT/mTOR activity, thus reducing the expression of genes involved in glycolytic pathways (Figure 5).

In addition to the above direct effects, many of these natural compounds also modify the TME. As shown in the above analysis, glycolysis in OC produces a large amount of lactate that is exported to the TME. Extracellular lactate promotes immune suppression and supports metastasis. Decreased glycolytic flux and lactate release lead to reduced extracellular acidification of these natural compounds. This may restore the function of CD8^+^ T cells and NK cells and suppress M2-like macrophage polarization. Some compounds, such as tanshinone I, also decrease histone lactylation (e.g., H3K18la) and thus reduce immune evasion by altering epigenetic marks. Agents including resveratrol and berberine have been shown to increase intra-tumoral CD4^+^ and CD8^+^ T-cell infiltration and decrease regulatory T cells and PD-L1 expression. Natural glycolysis inhibitors can, thus, inhibit the metabolism of cancer cells and promote the development of a favorable TME.

These natural compounds demonstrate significant potential in targeting the glycolytic pathway in OC, offering advantages such as multi-target effects, high safety profiles, and wide availability. Problems in extraction and purification, toxicity, bioavailability, and clinical application have not been addressed. One needs to be well-versed in the OC glycolytic pathway and know how to apply it in the treatment of diseases caused by natural products. These findings provide a comprehensive framework for understanding the multifaceted interactions between natural compounds and OC glycolysis, ultimately informing clinical treatment strategies and the development of novel therapeutic agents.

Natural compounds targeting glycolysis offer a novel approach to transforming the metabolic vulnerabilities of OC into a therapeutic advantage. These agents are individual treatment options and also the basis for combined treatment plans. At present, a multi-disciplinary approach is considered to be the most effective for expanding the anti-cancer effects of natural products. Technological progress has solved the problems of low bioavailability and instability of drugs. The development of advanced delivery systems—including nanoparticles, liposomes, and exosomes—allows for the encapsulation and modification of natural compounds. These platforms increase the water solubility and chemical stability of the drug and, at the same time, deliver it precisely to the lesion site in sufficient quantities for therapeutic effect.

Standardization of thethe extraction process and strict identification of the components is necessary to ensure batch-to-batch consistency and the reliability of research results for the next-generation of pharmaceutical products. Utilizing multi-omics technologies to elucidate the mechanisms of action of natural compounds, future research should focus on clarifying precise molecular targets and signaling pathways. For example, to understand how these compounds alter the upstream oncogenic signal of HIF-1α, c-Myc, and p53, one hopes to systematically reverse the Warburg effect in OC cells. Future work will also explore the effect of these compounds on lactate shuttling between CAFs and OC cells. Disruption of this metabolic cooperation will also harm the tumor. In addition, the aim of targeting monocarboxylate transporters (MCT1/MCT4) in combination with natural products may also be feasible. Good preclinical studies need to be carried out to test the safety and basic efficacy of a new drug. Establishing synergistic regimens that combine natural compounds with chemotherapy, targeted drugs, or immunotherapy offers alternative treatment options for patients with platinum-resistant recurrent OC. As these compounds can reduce lactate-driven immunosuppression, they may be used in conjunction with immune checkpoint inhibitors to overcome immune resistance in OC. A particular biomarker for the effect of treatment must be found, such as the expression level of a core glycolytic enzyme, and then the patients should be divided into two groups. Therefore, the treatment will be according to the specific gene mutations in a person. System biology and network pharmacology methods can be used to screen for and design synergistic, multi-component natural-compound combinations. These strategies can target different subtypes of OC selectively and offer a system for both general and individualized therapy.
